# MEX3A is a diagnostic, independent prognostic biomarker and a promising therapeutic target in glioblastoma

**DOI:** 10.3389/fonc.2025.1585592

**Published:** 2025-09-01

**Authors:** Francesca Bufalieri, Daniele Armocida, Antonino Cucinotta, Pietro Familiari, Laura Di Magno, Alessandra Serraino, Gennaro Adabbo, Francesca Agnoli, Ludovica Lospinoso Severini, Manila Antonelli, Alessandro Frati, Gianluca Canettieri, Paola Infante, Antonio Santoro, Luca D'Angelo, Lucia Di Marcotullio

**Affiliations:** ^1^ Department of Molecular Medicine, Sapienza University of Rome, Rome, Italy; ^2^ Experimental Neurosurgery Unit, IRCCS “Neuromed”, Pozzilli, Italy; ^3^ Neurosurgery Division, Human Neurosciences Department, Sapienza University of Rome, Rome, Italy; ^4^ Department of Radiological, Oncological and Anatomic Pathology Sciences, Sapienza University of Rome, Rome, Italy; ^5^ Istituto Pasteur-Fondazione Cenci Bolognetti, Sapienza University of Rome, Rome, Italy

**Keywords:** biomarker, gliomas, MEX3A, neuro-oncology, therapeutic target

## Abstract

**Objective:**

Gliomas are the most common malignant brain tumors with a poor prognosis. Despite advances in molecular profiling, no targeted therapies significantly improve survival. Recently, it has been demonstrated that high expression of Muscle Excess 3A (MEX3A) correlates with poor overall survival (OS) in gliomas, generating interest in its potential as a biomarker and therapeutic target. This study analyzes the correlation between *MEX3A* expression and clinical-molecular features, assessing its diagnostic, prognostic, and therapeutic value in glioblastoma (GB), the most aggressive glioma subtype.

**Methods:**

We performed a retrospective study on a consecutive series of surgically-treated glioma patients. The values of *MEX3A* mRNA levels for the discrete variables examined has been reported by boxplots. Chi-square tests were carried out to analyze the correlation between *MEX3A* expression and patient features. Receiver operating characteristic (ROC) curve, Kaplan-Meier survival and Cox regression analysis were applied to assess the diagnostic and independent prognostic values of MEX3A in GB. Finally, the effect of *MEX3A* genetic knockdown on human primary GB both *in vitro* and *in vivo* orthotopic xenograft model cell has been evaluated.

**Results:**

Elevated MEX3A expression associates with more severe clinicopathological and molecular features of glioma patients. MEX3A exhibits high diagnostic accuracy (AUC > 0.9) and correlates with poor OS (HR=2.068, p=0.0018) and progression-free survival (PFS) (HR=2.209, p=0.0005) in GB. Multivariate Cox regression identified MEX3A as an independent prognostic factor for OS and PFS. Notably, *MEX3A* knockdown inhibits tumor growth *in vitro* and *in vivo.*

**Conclusions:**

Our findings highlight MEX3A as a novel diagnostic and prognostic biomarker and a promising therapeutic target for GB.

## Introduction

Cancer remains one of the leading causes of death worldwide, with an increasing global burden despite significant advances in diagnosis and therapy (1). Among human malignancies brain tumors represent a particularly devastating category due to their complex biology, limited treatment options, and poor prognosis. Gliomas, especially in their most aggressive form glioblastoma (GB), continue to be lethal and incurable tumors (2, 3). Gliomas account for about 30%of all primary central nervous system (CNS) malignancies and about 80% of all malignant brain tumors, with an average mortality rate of 4.41 per 100.000 and 17.411 deaths per year (3–6).

The standard management consists of a multidisciplinary approach, including surgical tumor mass resection, radiotherapy, and chemotherapy with Temozolomide (TMZ) (7). Despite these therapeutic regimens, a high rate of relapse is observed in treated patients mainly due to the molecular heterogeneity of these tumors, often coupled with a decline in neurological function and quality of life (8, 9). New technologies, including next-generation sequencing and advanced statistical tools (10), allowed to create a new and more detailed World Health Organization (WHO) classification of gliomas with different biomarkers for each specific molecular profiling (11, 12). The major factors routinely assessed for gliomas studies include mutation in isocitrate dehydrogenase 1 (*IDH1*), the expression and mutation of the epidermal growth factor receptor (*EGFR*), the cell replication index reported as percentage of Ki67 expression, and the O^6^-methylguanine-DNA-methyltransferase (*MGMT*) promoter hypermethylation, the major predictive factor for treatment response to TMZ in GB patients (13, 14). The fifth edition of the WHO classification of tumors of the CNS released in 2021, have led to a more biologically homogeneous categorization of gliomas into three types: astrocytoma, (IDH)-mutant (grade 2,3,4); oligodendroglioma, IDH-mutant and 1p/19q-codeleted (grade 2,3); GB, IDH-wildtype (grade 4) (4). *IDH*-wildtype GB accounts for nearly 90% of all grade IV gliomas, with a median survival of just 15 months and only 5.5% of patients surviving beyond 5 years post-diagnosis (2–4, 12). Despite promising preclinical results, no therapies targeting the biomarkers identified so far significantly increase the survival rate of GB patients (15, 16). As result, the lack of effective treatments highlights the pressing need to discover new biomarkers that might be exploited for a more accurate diagnosis and prognosis, as well as to develop personalized targeted therapies.

Recent studies revealed that the Muscle Excess 3 (MEX3) protein family is expressed across various cancers (17), playing a role in regulating numerous oncogenic processes, such as tumor cell self-renewal (18). First discovered in *Caenorhabditis elegans*, the MEX3 proteins are evolutionarily conserved in mice and mammals and consist of four members (MEX3A, MEX3B, MEX3C, and MEX3D) (18). In agreement with their domain composition, MEX3 proteins bind RNAs, modulating their fate, and work as E3 ubiquitin ligases that affect the stability and the subcellular localization of their specific protein substrates (18). Among the MEX3 family members, MEX3A has been found overexpressed in several human malignancies and recently it has emerged as a promising biomarker and therapeutic target in a wide range of cancers (19–25). Although the involvement of MEX3A in glioma pathogenesis and resistance to treatments has previously been explored (23, 26, 27), its clinical relevance deserves further investigations.

Here, we analyze the association of high expression levels of MEX3A with clinical and molecular aspects of a consecutive series of surgically treated patients suffering from intracranial gliomas and we discover the value of MEX3A as a diagnostic and prognostic independent factor in GB patients. Moreover, we demonstrate that the inhibition of MEX3A arrests tumor growth *in vitro* and *in vivo*. Our findings highlight MEX3A as a practical tool for predicting the diagnosis and prognosis of GB patients and unveil its potential as innovative target for tailored therapeutic options.

## Methods

### Clinical samples

This retrospective observational study was performed on a surgical series of glioma patients treated in a single neurosurgical unit. Consensus about diagnosis, treatment, and related information was obtained under written informed consent approved by our Institution’s Principal Institutional Review Board (IRB: 6961, prot. 0296/2023). This study adheres to PROBE 2023 guidelines for reporting observational studies. All methods were carried out following relevant guidelines and regulations.

Data from adult patients who underwent surgery for glioma in our institution between January 2020 and December 2022 were analyzed. Patients were enrolled according to the following criteria: age >18 years; preoperative magnetic resonance imaging (MRI) is suggestive for glioma; no previous surgery; no previous radiotherapy; at least 18 months of follow-up; patients that undergo a standard STUPP protocol (7) starting from the 30^th^ day after surgery.

Patients were excluded if they had a histologic diagnosis other than glioma, had not fully performed therapeutic or diagnostic follow-up, and had radiological material not available on PACS.

All the patients included underwent a preoperative brain MRI scan included a high field 3 Tesla volumetric study with the following sequences: T2w, FLAIR, isotropic volumetric T1-weighted magnetization-prepared rapid acquisition gradient echo (MP-RAGE) before and after intravenous administration of paramagnetic contrast agent (28, 29).

All participating patients underwent surgery to completely diagnose and remove the tumor mass. The procedures were carried out using an infrared-based Neuronavigator (Brainlab, Kick^®^ Purely Navigation) in a standard neurosurgical theater, equipped with a standard operative microscope (Leica, model OH4). Our institution’s surgical protocol was followed (30), where the extent of resection (EOR) was deemed complete when the white matter was free of disease in all aspects of the surgical cavity. The excision was discontinued by the surgical operator when, despite direct visualization or navigation remnants, neuromonitoring or intraoperative neuropsychological testing indicated a potential for postoperative motor complications.

In the postoperative day, patients underwent a CT-scan to assess major early complications and a volumetric Brain MRI scan to evaluate the EOR. For gross total resection (GTR), “tumor progression” was defined as the initial MRI scan showing the presence of pathologically enhancing tissue, characterized by an MRI pattern inconsistent with cerebral radiation injury, which is considered a “pseudo-progression.” In the case of incomplete resections (<95% volume reduction), a volumetric increase of the residual disease detected at the first postoperative MRI scan was considered as disease progression, and this was used to calculate the PFS. Our institution had a dedicated neuro-imaging follow-up program that included a standard early (maximum 24 hours after surgery) postoperative volumetric brain MRI, a volumetric brain MRI scan repeated at one month from surgery (25–35 days) for the first step follow-up control to provide information for the radiation treatment planning, and a volumetric brain MRI scan performed every three months.

### Clinical and pathological analysis

For all the included patients we recorded age, gender, IDH R132H, Ki67, P53 and EGFR expression status. The expression of IDH1 R132H, P53, EGFR and Ki67 in formalin-fixed paraffin embedded (FFPE) tumor tissues was analyzed by standard immunohistochemistry (IHC) technique carried out in the Department of Neuropathology of our University Hospital (31). The following antibody were used: anti-P53 (DO-7, 1:40; Cell Marque, Hot Springs, AZ, USA), which detect both wild type and mutant P53 protein (28); anti-IDH1 R132H (DIA-H09, 1:50; Dianova, Hamburg, Germany); anti-EGFR (clone H11; 1:200; Dako, Glostrup, Denmark); anti Ki67 (MIB-1, 1:50; Dako, Glostrup, Denmark) (32). Following counterstaining with hematoxylin, slides were dehydrated, mounted and observed under light microscope. At least 200 tumor cells from different fields (from 5 to 10) were reviewed. Scoring was performed by semi-quantitative scoring, independent to diagnosis, with not-expressed/negative (no staining observed), expressed (up to 50% cells are stained) and highly expressed (>50% of cells stained). %Ki67 was measured with the “hot spot method” where the field with highest apparent Ki67 index was selected and up to 500 cells scored (33).

Histological diagnoses were performed according to the updated version of the 2021 WHO guidelines (4). PFS and OS was recorded in months; it was measured from date of diagnosis to date of death or date of last contact if alive. Clinical information was obtained by the digital database of our Institution, whereas OS data, were obtained by telephone-interview.

### Cell cultures and lentiviral infection

Primary GB-derived neurosphere cultures were obtained after mechanical dissociation from high-grade gliomas freshly resected from patients. In brief, the tissue was first washed in HBSS plus penicillin–streptomycin (1%) to remove excess debris and blood, and the tumor has been cut and mechanically minced before digested with deoxyribonuclease. The digested tissue was titrated and passed through a cell strainer. Finally, cells were pelleted by centrifugation (300 X *g* for 10 min) and cultured in Neurobasal medium supplemented with B27 without vitamin A (2%), penicillin–streptomycin (1%), L-glutamine (1%), N-Acetyl-L-Cysteine (60 ng/ml) human EGF (20ng/ml) and human FGF (20ng/ml).

All the GB cell lines were validated by short tandem repeat (STR) DNA profiling performed by Eurofins Genomic Europe (Ebersberg, Germany) in November 2023 and preserved in liquid nitrogen to preserve authenticity. Mycoplasma contamination in cell cultures was routinely detected by using PCR detection kit (Applied Biological Materials, Richmond, BC, Canada).

For *MEX3A* genetic depletion, lentiviral particles were generated in HEK293 cells transfected with packaging and envelope plasmids (pCMV-dR8.74 and VSV-G/pMDG2), pGFP-pLKO.1 plasmids (shCTR TR30021; shMEX3A TL308061B (#1), TL308061C (#2), Origene, Rockville, MD, USA). GB tumor spheroids were dissociated in cell dissociation solution (C5789, Merck, Darmstadt, Germany) and GB6 cells were infected with purified lentiviral particles resuspended in complete medium for 72 h.

For animal study, GB6 cells were infected with both lentiviral particles expressing shCTR or shMEX3A #2 and the luciferase reporter (pLenti CMV Puro Luc w168-1, Addgene, Watertown, Massachusetts, USA), generated as described above.

### mRNA expression analysis

Total RNA was isolated from gliomas tissues and peritumoral brain normal tissues, using TRIzol reagent (Invitrogen/Life Technologies, Carlsbad, CA, USA), and reverse-transcribed with a SensiFAST cDNA Synthesis Kit (Bioline Reagents Limited, London, UK). Quantitative real-time PCR (qRT-PCR) analysis of mRNA expression for the indicated genes was performed by using the ViiATM 7 Real-Time PCR System (Life Technologies). A reaction mixture containing cDNA template, SensiFAST™ Probe Lo-ROX mix (Bioline Reagents Limited) and Taqman Gene Expression Assays (Thermo Fisher Scientific Waltham, MA, US) was amplified using standard qPCR thermal cycler parameters and mRNA quantification was performed by using SDS version 2.3 software. Each sample was amplified in triplicate, and the average of the three threshold cycles was used to calculate the number of transcripts. Data were normalized to the endogenous controls (*GAPDH* and *HPRT*), which yielded similar results. And expressed as the fold change respect to the control sample value.

To compare the mRNA expression of *MEX3A* between the patients samples we considered the value of 2ˆ^(-ΔΔCT)^. For the *in vitro* and *in vivo* experiments in primary gliomas cell lines mRNA expression of the indicated genes was expressed as the fold change respect to the control sample value.

The following TaqMan Gene Expression Assays (Thermo Fisher Scientific) were used: *MEX3A*, Hs00863536_m1; *NANOG*, Hs02387400_g1; *POU5F1*, Hs04260367_gH; *SOX2*, Hs04234036_s1;*GAPDH*, Hs02786624_g1;*HPRT*, Hs02800695_m1.

### Immunoblot analysis

For immunoblot analysis, cells were lysed in RIPA buffer (50 mM Tris-HCl pH 7.6, 150 mM NaCl, 0.5% sodium deoxycholate, 5 mM EDTA, 0.1% SDS, 100 mM NaF, 2 mM NaPPi, and 1% NP-40) supplemented with protease and phosphatase inhibitors. Lysates were incubated on ice and then centrifuged at 13,000 × g for 30 minutes at 4°C. Following centrifugation, a defined volume of the supernatant was mixed with sample loading buffer, boiled for 5 minutes, resolved by SDS-PAGE, and subjected to immunoblot analysis. The mouse monoclonal anti-β-Actin antibody C4 (sc-47778, 1:2000) was purchased from Santa Cruz Biotechnology (Santa Cruz, CA, USA); Rabbit polyclonal anti-MEX3A antibody (ab79046, 1:1000) was purchased from Abcam (Cambridge, UK); rabbit EGF receptor D381B1 antibody (4267S, 1:1000), mouse OCT4 D705Z antibody (7543S, 1:1000), rabbit Vimentin R2B antibody (3932S, 1:2000) were purchased from Cell Signaling (Beverly, MA, USA); rabbit E-Cadherin antibody (20874-1-AP, 1:3000) was purchased from Proteintech (Thermo Fisher Scientific, Waltham, MA, USA). HRP-conjugated secondary antibodies were purchased from Bethyl Laboratories (Montgomery, TX, USA).

### Immunohistochemistry

Tumor and peritumoral tissues from patients and brain tissues from the orthotopic GB6 implanted mouse were fixed in formalin and paraffin-embedded (FFPE) and cut into 5μm sections FFPE slides were deparaffinized and subjected to heat-induced antigen retrieval at low or high pH buffer and blocked for 30 min with 5% PBS/BSA. Then, patient-derived slides were incubated with the anti-MEX3A (Rabbit anti-MEX3A ab79046, 1:100) antibody, whereas slides from the orthotopic model were incubated with antibodies against MEX3A, Ki67 (Rabbit anti-Ki67 SP6, MA5-14520, 1:100; Thermo Fisher Scientific, Waltham, MA, US) and SOX2 (Rabbit-anti SOX2 ab97959, 1:100; Abcam, Cambridge, UK).

The day after, the slides were incubated for 20 min with secondary antibodies coupled with peroxidase (Dako, Glostrup, Denmark). Bound peroxidase was detected with diaminobenzidine (DAB) solution and EnVision FLEX Substrate buffer containing peroxide (Dako, Glostrup, Denmark). After counterstaining with hematoxylin, sections were dehydrated in a graded water-ethanol series, mounted and observed under light microscope. Cell quantification was performed on collected sections using the imaging software NIS-Elements BR 4.00.05 (Nikon Instruments Europe B.V., Italy).

### Cell proliferation assay

For IncuCyte^®^ experiments, infected GB cells were seeded in 96-well plates (20 x10^3^ cells/well for each cell lines; 12 wells for each condition) in complete medium and treated with IncuCyte^®^ NucLight Rapid Red Reagent (#4717, Essen BioScience, 1:1000). Plates were transferred into the IncuCyte^®^ S3 Live Cell Analysis Systems and incubated at physiological conditions (37°C, 5% CO_2_), over 96 h. Images were collected every 6 h, and proliferation was evaluated as the ratio number of infected cells on NucLight^®^ positive cells. The experiments were performed in triplicate, and data were analyzed by using the IncuCyte^®^ software package (Essen BioScience, Ann Arbor, MI, USA).

### GB-derived neurospheres formation assay

Dissociated primary GB cells for each experimental groups (shCTR, shMEX3A#1 and shMEX3A#2) were plated in 96-well plates in decreasing numbers (50, 25, 10, 5 cells; 12 well for each condition, 12 replicates per cell/density number) in neurospheres culture media. The plates were incubated in a 37°C, 5% CO_2,_ humidified incubator. At ten days, any well that contains neurospheres was scored. Extreme limiting dilution analysis was conducted using the software available at http://bioinf.wehi.Edu.au/software/elda/.

### Orthotopic xenograft study

Female NOD/SCID gamma (NSC) mice (6 weeks old) were used. All described procedures involving experimental animals were performed in agreement with standard guidelines under a protocol approved by local ethic authorities (Ministry of Health) and conducted in accordance with Italian Governing Law (D.lgs 26/2014).

For establishing intracranial GB, 3 x 10^5^ cells from GB6 cell line transduced with lentiviral particles expressing shMEX3A#2 or shCTR, and the luciferase reporter, were stereotaxically implanted into the striatum of the mice device (coordinates: 2 mm anterior, 2 mm lateral, 3 mm depth from the dura).

Tumors were analyzed by luminescence imaging (IVIS Lumina III, PerkinElmer, USA) each week. Before imaging, mouse underwent an intraperitoneal injection of D-luciferin (10 μl g^−1^, XenoLight RediJect D-Luciferin, PerkinElmer, USA). Mice were sacrificed upon signs of tumor formation (weight loss, hunching, rough coat, level of consciousness and activity) and brains were fixed in 4% formaldehyde paraffin embedded and processed for histological and IHC analysis. Hematoxylin and eosin (H&E) staining was performed and reviewed by board-certified pathologists, who confirmed GB-like histopathological features.

### Statistical methods

Statistical analyses were performed with GraphPad Prism software version 9.5.1 (GraphPad, San Diego, CA, USA). FFPE brain tissues from the orthotopic GB6 implanted mouse were cut into 5μm sections for MEX3A, Ki67 and sex determining region Y-box2 (SOX2) immunohistochemical staining. FFPE slides were deparaffinized and subjected to heat-induced antigen retrieval at low or high pH buffer and blocked for 30 min with 5% PBS/BSA. Then, the slides were incubated overnight at 4°C with monoclonal antibodies against MEX3A Figure(Rabbit anti-MEX3A ab79046, 1:100; Abcam, Cambridge, UK), Ki67 (Rabbit anti-Ki67 SP6, MA5-14520, 1:100; Thermo Fisher Scientific, Waltham, MA, US) and SOX2 (Rabbit-anti SOX2 ab97959,:100; Abcam, Cambridge, UK) (19, 20, 23–25). MEX3A expression in different groups was compared using two-tailed Student’s *t* test or one-way ANOVA. The association between MEX3A expression and clinicopathological and molecular characteristics of the patients was assessed by χ^2^ test for assessing the association between two categorical variables (19, 20, 23–25, 34).

The sensitivity and specificity of MEX3A in *IDH*-wildtype GB diagnosis was evaluated using receiver operating characteristic (ROC) curve. Survival curves of the same patients were carried out using Kaplan-Meyer method, assessing differences in OS and PFS between the indicated groups by log-rank test. Univariate and multivariate survival analysis were performed using the Cox proportional hazard regression model. Only the features with prognostic significance in univariate analysis were included in the subsequent multivariate analysis. We used a significance threshold of P < 0.05, which is the conventional alpha level in biomedical research for determining statistical significance (19, 20, 23–25, 35). The threshold of statistical significance was considered *p* < 0.05.

### Potential source of bias and study size

A potential source of bias is expected from the exiguity of the sample, which, nevertheless, in regard to the endpoints selected, presents an excellent *post-hoc* statistical estimated power (1- β = 0.9402 for α 0.05 and effect size “f” = 0.34), thus providing extremely reliable conclusions.

## Results

### Clinical characteristics of patients

From the initial group of 156 screened patients, 24 were excluded: 8 because their final diagnosis was not glioma, 6 due to their inability or refusal to give consent, and 10 because of insufficient biological samples. After the initial screening, 29 more patients were excluded from the study: 6 for not following standard follow-up protocols and 23 due to discrepancies in surgical documentation. Additionally, 22 patients were lost to follow-up (**Supplementary Figure S1**).

The final cohort comprised 81 patients with histologically diagnosed glioma, 53 males and 28 females. The mean survival identified was 14.7 months (min 1, max 53), with a mean progression free survival (PFS) of 6.8 months (min 1, max 32). 12 patients were alive at the last follow-up visit (June 2024) and 9 patients had no presence of disease progression. The most frequent histologic diagnosis was GB (63 patients, 77.8% of cases), followed by diffuse astrocytoma (7 patients, 8.6% of cases), anaplastic astrocytoma and oligodendroglioma (4 patients each, 4.9% of cases), gliosarcoma (2 patients, 2.5% of cases) and one case of polymorphous neuroepithelial tumor in the young (PLTNY, 1.2% of cases). Clinical and molecular data are summarized in **Supplementary Table S1**.

### The overexpression of MEX3A associates with clinicopathological and molecular features of gliomas patients

We investigated the putative association between mRNA expression levels of *MEX3A* and several clinicopathological and molecular characteristics of glioma patients. Grouping *MEX3A* expression by different variables, we observed that higher levels of *MEX3A* correlate with features of poor prognosis such as older age, EGFR and Ki67 high expression (**Supplementary Figures S2A, F, H**, respectively). Accordingly, *MEX3A* expression increases with higher tumor grade (**Supplementary Figure S2C**) and was significantly associated with vital status (**Supplementary Figure S2D**). On the contrary, lower levels of *MEX3A* were observed in *IDH1* mutated gliomas (**Supplementary Figure S2E**). No significant association was found between *MEX3A* expression and other clinicopathological parameters, including patients’ gender and P53 expression (**Supplementary Figures S2B,G**, respectively). Finally, we divided all glioma patients into high and low *MEX3A* mRNA expression groups using the median expression level as a cut-off. Chi-square analysis of the variables between the two groups confirmed the association of *MEX3A* expression with the clinical and molecular characteristics of the analyzed patients (**Table 1**).

**Table 1:** Correlation between *MEX3A* mRNA expression and clinical and molecular characteristics of glioma patients.

Clinical features		Number of patients			Statistical parameters	
Variable	Covariate	Total (81)	Low MEX3A expression (39)	High MEX3A expression (42)	χ^2^	p-value
Age	≤ 64	40	25	15	5.440	**0.0197**
	>64	41	15	26		
Gender	Male	53	27	26	0.149	0.699
	Female	28	13	15		
Grade	I-II	7	7	0	7.676	**0.0215**
	III	7	4	3		
	IV	67	28	34		
Vital status	Alive	12	9	3	5.505	**0.025**
	Dead	65	26	39		
IDH1 (R132H)	Not mutated	69	30	39	8.538	**0.0035**
	Mutated	11	10	1		
EGFR	Not expressed	16	11	5	8.421	**0.0148**
	Expressed	14	8	6		
	Highly expressed	27	7	20		
P53	Not expressed	39	23	16	2.102	0.147
	Expressed	42	18	24		
Ki67 (%)	<20%	29	21	8	9.277	**0.0023**
	≥20%	49	18	31		

p< 0.05 is marked in bold.

### MEX3A expression has a diagnostic and prognostic value in GB

**Figure 1:**
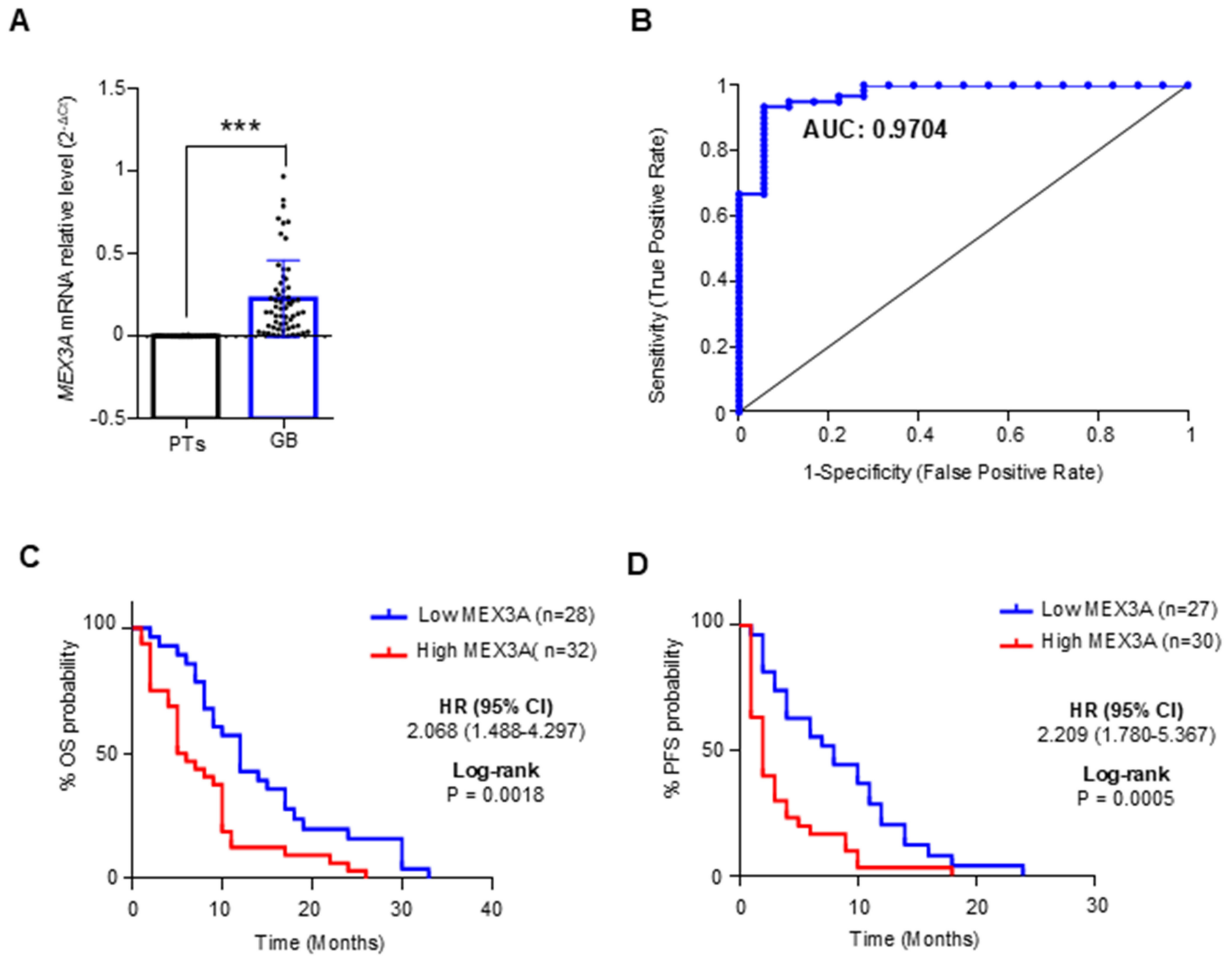
Diagnostic and prognostic value of MEX3A in *IDH*-wildtype GB (GB). **(A)** *MEX3A* mRNA expression in 63 cases of GB compared to 18 peritumoral tissues (PTs). Mean ± SD; P*** < 0.001. **(B)** ROC curve for *MEX3A* mRNA expression in GB and PTs shown in **(A)** AUC= 0.9704. **(C, D)** Kaplan-Meyer curves for OS **(C)** and PFS **(D)** between high and low mRNA expression groups of *MEX3A*.

We evaluated the diagnostic and prognostic potential of MEX3Ain *IDH*-wildtype glioblastoma (hereafter referred to as GB), since this group represents the most common and malignant variant among gliomas. First, we confirmed the up-regulation of *MEX3A* transcript in GB patients compared to the peritumoral brain tissues (PTs) (**Figure 1A**), and next we evaluated the diagnostic value of *MEX3A* expression for these patients. ROC curve was generated to discriminate *MEX3A* expression in GB tissues from the PTs. We found that the area under curve (AUC) for *MEX3A* was 0.9704 (95% CI: 0.9310-1.010; p<0.0001) (**Figure 1B**), indicating that *MEX3A* expression has a high sensitivity and specificity for GB diagnosis.

To assess the prognostic potential of MEX3A for OS and progression free survival (PFS), GB samples were divided into two groups based on their median *MEX3A* mRNA expression levels. Kaplan Meyer analysis revealed that higher expression of *MEX3A* Figurewas significantly related to shorter OS (HR=2.068, *p*=0.0018) and PFS (HR=2.209, *p*=0.0005), suggesting that *MEX3A* has also a prognostic value in GB patients (**Figures 1C, D**). Importantly, these findings were further validated at protein level by IHC analysis performed on the available patient-derived GB and peritumoral tissue samples, confirming the elevated expression of MEX3A in tumor tissues, and its diagnostic and prognostic potential in GB (**Supplementary Figure S3**).

### MEX3A is an independent predictor for OS and PFS for GB patients

**Figure 2:**
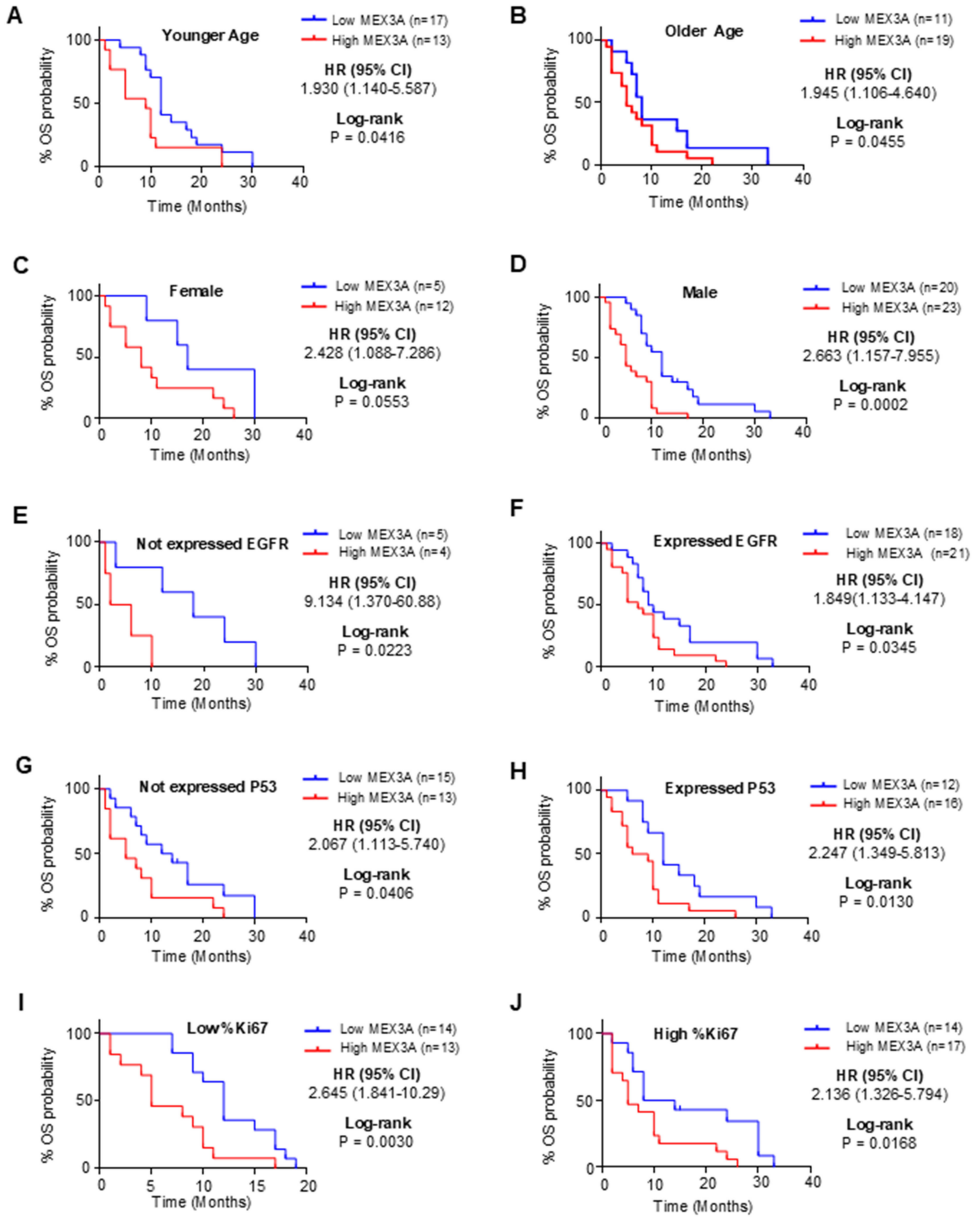
Analysis of OS between high and low mRNA expression groups of *MEX3A* according to the different clinical variables of *IDH*-wildtype GB (GB) patients. Subgroup analysis was performed in young patients **(A)**, old patients **(B)**, females **(C)**, males **(D)**, patients with no EGFR expression **(E)**, patients with EGFR expression **(F)**, patients with no P53 expression **(G)**, patients with P53 expression **(H)**, patients with low %Ki67 expression **(I)** and patients with low %Ki67 expression **(J)**.

**Figure 3:**
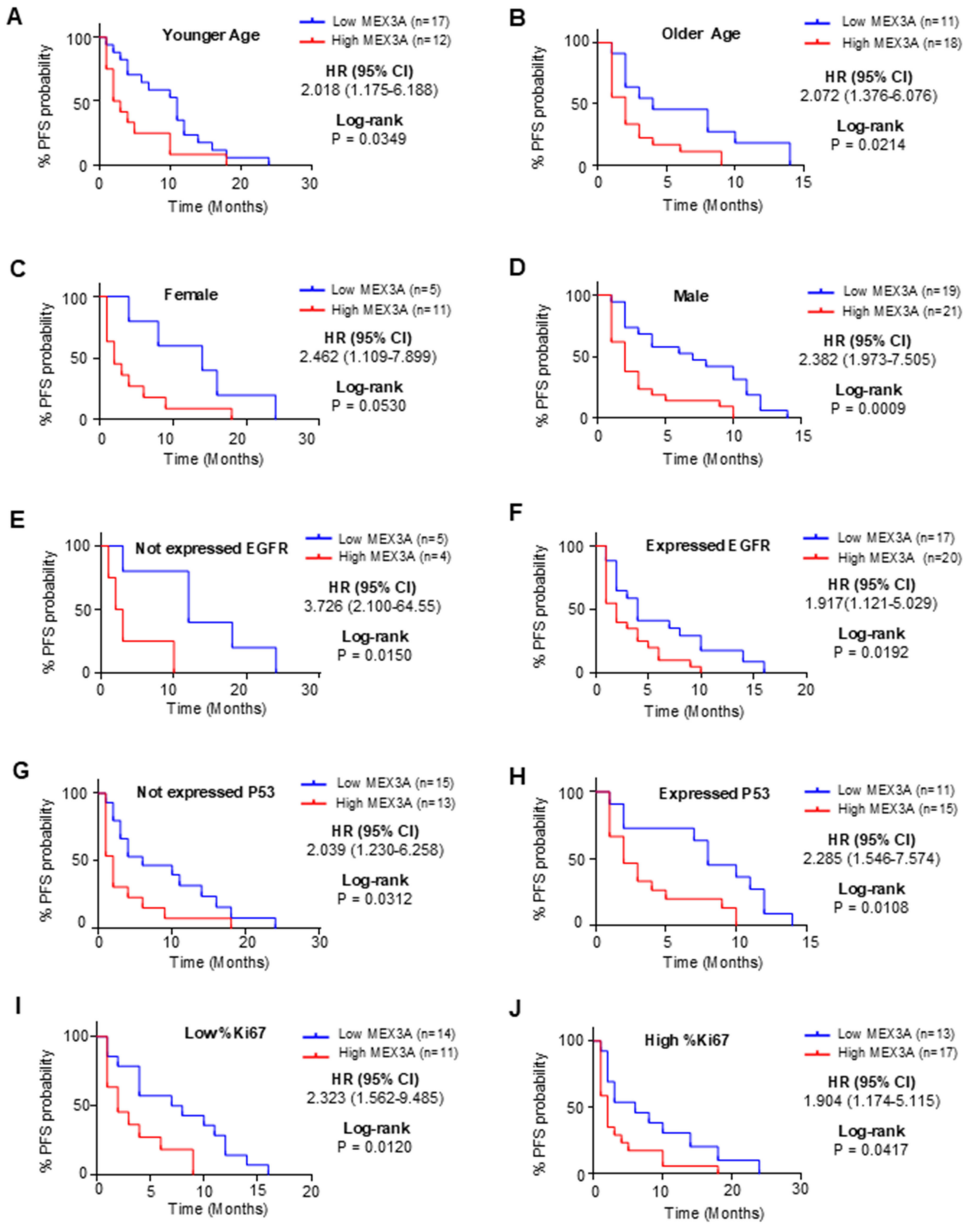
Analysis of PFS between high and low mRNA expression groups of *MEX3A* according to the different clinical variables of *IDH*-wildtype GB (GB) patients. Subgroup analysis was performed in young patients **(A)**, old patients **(B)**, females **(C)**, males **(D)**, patients with no EGFR expression **(E)**, patients with EGFR expression **(F)**, patients with no P53 expression **(G)**, patients with P53 expression **(H)**, patients with low %Ki67 expression **(I)** and patients with low %Ki67 expression **(J)**.

To further explore the prognostic value of MEX3A, we assessed its association with clinical outcomes across multiple subgroups. High *MEX3A* mRNA expression was significantly associated with poorer OS (**Figure 2**) and PFS (**Figure 3**) in all examined clinicopathological variables, including age, gender, and the expression levels of EGFR, P53, and Ki67. Moreover to determine whether MEX3A expression act as an independent prognostic factor for the OS and PFS for GB patients, univariate and multivariate COX regression analysis has been performed. We found that the high expression of *MEX3A* is an independent predictor for OS (HR: 3.368; 95% CI 1.680-6.969; p = 0.0008), as well as for PFS (HR: 2.456; 95% CI 1.340-4.574; p = 0.004) in our cohort of GB patients (**Table 2**). These findings are consistent with the oncogenic role of MEX3A observed in other tumor types, where MEX3A has been shown to promote cancer progression by regulating RNA stability, activating signaling pathways involved in stemness and proliferation, and contributing to therapy resistance (19–25).

**Table 2:** Univariate and multivariate COX regression analysis for OS and PFS.

Variable	Outcome	Univariate analysis		Multivariate analysis	
		*P* value	HR (95% CI)	*P* value	HR (95% CI)
MEX3A (High, Low)	OS	**0.0020**	2.373 (1.378-4.149)	**0.0008**	3.368 (1.680-6.969)
	PFS	**0.0011**	2.510 (1.447-4.397)	**0.0040**	2.456 (1.340-4.574)
Age (≤ 65; >65)	OS	0.2567	1.354 (0.7979-2.289)		
	PFS	**0.0007**	2.887 (1.579-5.391)	**0.0008**	3.007 (1.593-5.778)
Sex (F, M)	OS	0.1018	1.636 (0.9237-3.026)		
	PFS	0.5427	1.198 (0.6801- 2.193)		
EGFR expression (Yes, No)	OS	0.6457	1.175(0.6095- 2.450)		
	PFS	0.0859	1.673(0.9457- 3.084)		
P53 expression (Yes, No)	OS	0.7818	1.076 (0.6377-1.819)		
	PFS	0.3662	0.7755 (0.4441-1.347)		
%Ki67 (<25%, ≥25%)	OS	0.185	0.6797 (0.3812-1.203)		
	PFS	0.9241	1.027 (0.5889-1.795)		

p< 0.05 is marked in bold.

### Genetic silencing of MEX3A significantly impairs primary human GB-derived neurospheres growth

To further explore the oncogenic role of MEX3A in GB, we established several primary human GB-derived neurospheres lines (**Supplementary Table S2**). Cancer-derived spheroids have the ability to maintain the heterogeneity of the original tumor (**Supplementary Figure S4**), showing self-renewal capacity, enhanced tumor-initiating, and tumor-propagating properties, mirroring the multipotency of glioma stem cells (GSCs) (36, 37).

**Figure 4:**
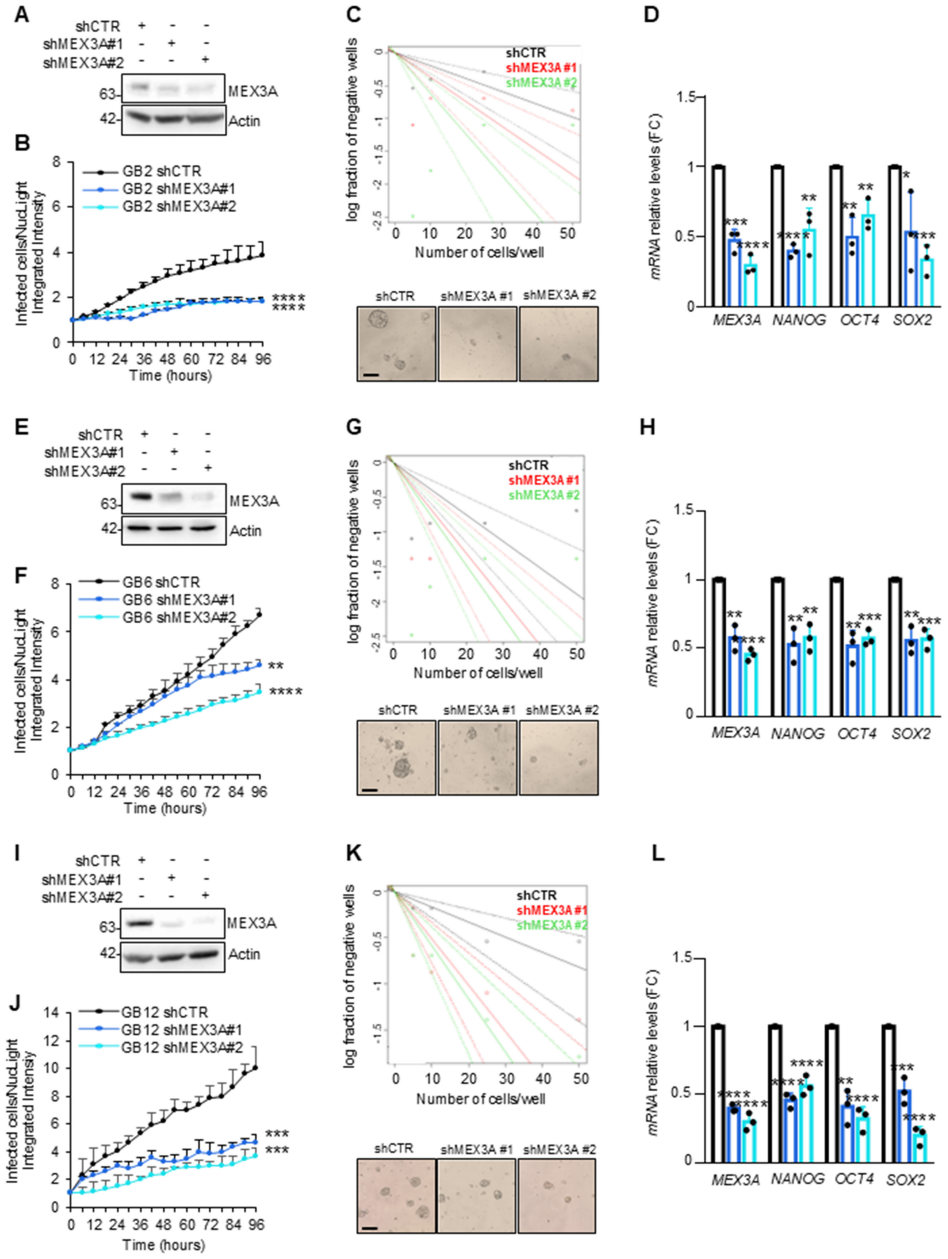
Effect of *MEX3A* genetic depletion on primary GB-derived neurospheres. **(A, E, I)** Immunoblot analysis of MEX3A in GB2, GB6 and GB12 primary GB-derived neurospheres (reported in **Supplementary Table S2**) following infection with lentiviral particles encoding either control shRNA (shCTR) or MEX3A shRNAs (shMEX3A#1 and shMEX3A#2). **(B, F, J)** Primary GB cells proliferation was measured as fold change (FC) of infected cells on NucLight positive cells calculated by fluorescent live cells imaging using IncuCyte Zoom software. **(C, G, K)** Limiting dilution assay performed in GB-derived neurospheres genetically depleted for *MEX3A*. Representative bright field images of GB-derived neurospheres formation capacity was shown. Scale bar: 100 μm. **(D, H, L)** qRT-PCR analysis of *MEX3A* and the indicated stemness genes in GB2, GB6 and GB12 cells after *MEX3A* genetic depletion. Data are normalized to endogenous *GAPDH* and *HPRT* controls and expressed as the fold change respect to the control sample value. Data represent the mean of three independent experiments. Mean ± SD. *P < 0.05; **P < 0.01; ***P < 0.001; ****P < 0.0001 calculated by two-sided Student’s *t*-test.

GB-derived neurospheres from GB samples with different molecular backgrounds (**Supplementary Table S2**) were genetically silenced for *MEX3A* by infection with lentiviral particles encoding two different short hairpins RNA or non-targeting shRNA as control (**Figures 4A, E, I**). Interestingly, regardless of the molecular characteristics, the proliferation and clonogenic self-renewal ability of GB-derived neurospheres was strongly impaired upon *MEX3A* knockdown (**Figures 4B, F, J, C, G, K**, respectively). Moreover, MEX3A silencing also led to a reduction in the expression of the invasion marker vimentin, accompanied by an increase in the adhesion marker E-cadherin (**Supplementary Figure S5**). Finally, given *MEX3A* function as stemness gene (38, 39), we observed a concomitant reduced expression of the well-known stemness markers *OCT4*, *NANOG*, and *SOX2* (**Figures 4D, H, L**) suggesting a crucial role of MEX3A in maintaining the stem-like properties of GB cells.

### MEX3A knockdown inhibits GB growth in vivo and prolongs mice survival

**Figure 5:**
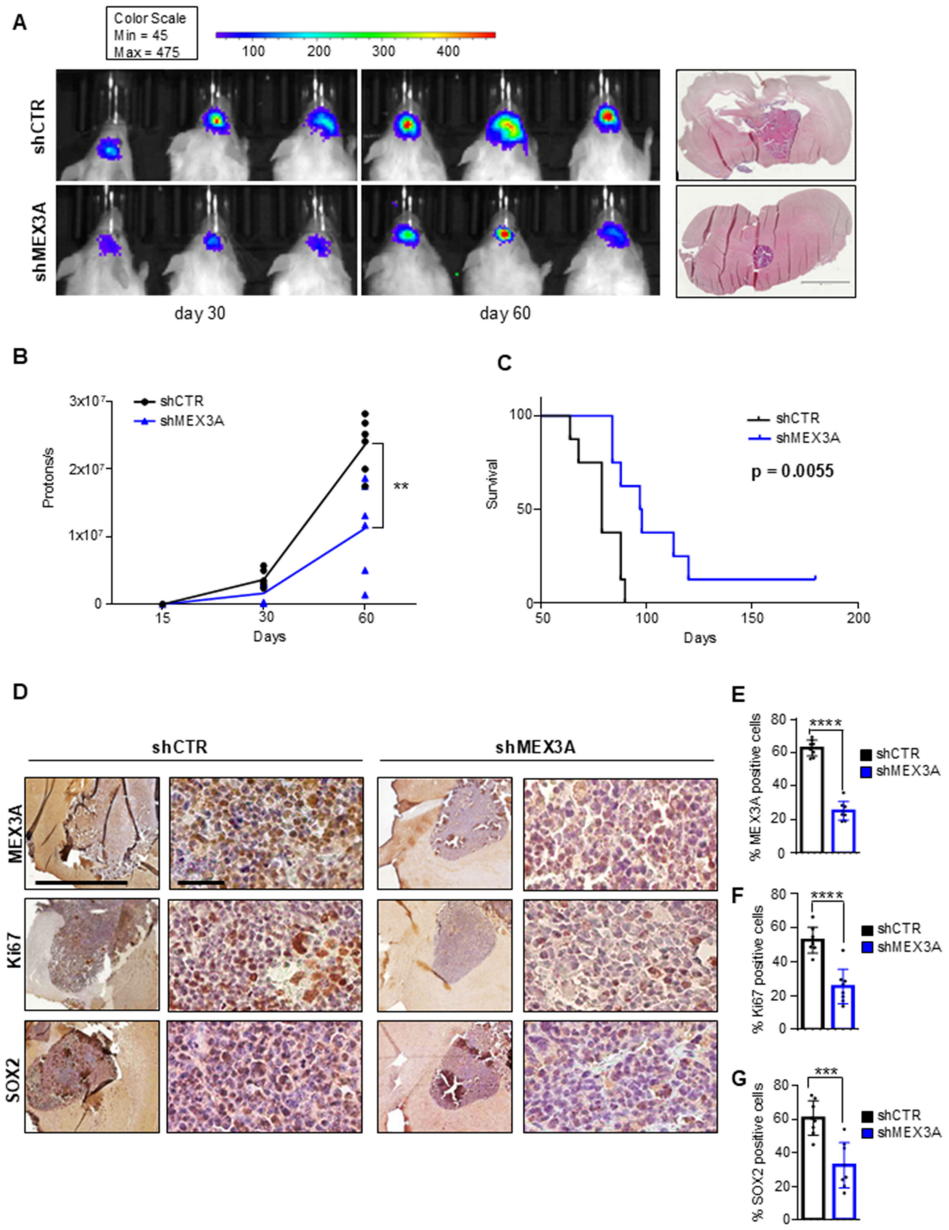
MEX3A inhibition impairs GB tumor growth *in vivo.* GB6 primary cells transduced with lentiviral particles encoding shCTR or shMEX3A and expressing luciferin were injected into the brain of NSG mice (n=8 for each group). **(A)** Pseudocolor representation of bioluminescence signal of intracranial xenografts bearing GB6 primary cells stably expressing shCTR or shMEX3A at the indicated days. Representative hematoxylin and eosin (H&E) staining for tumor brain section was shown on the right. Scale bar 2mm. **(B)** Quantitative analysis of luciferase activity in mice forming tumor from MEX3A-depleted cells or control cells at 15, 30, and 60 days after implantation. Data are shown as individual values plotted. Statistical analysis was performed using two-way ANOVA. The effect of the sample group was statistically significant, accounting for 7.09% of the total variance (after adjusting for matching; F (1,10) = 19.74, **P = 0.0012). **(C)** Survival curve of mice with GB cell-derived orthotopic tumor genetically interfered for MEX3A compared to control group. **(D)** MEX3A, Ki67 and SOX2 immunohistochemical staining of tumor samples. Scale bars 2mm and 50 µM. **(E-G)** Quantification of immunohistochemical staining shown in **(D)**. Mean ± SD; ***P < 0.001; ****P < 0.0001 calculated by two-sided Student’s *t*-test.

Given the potency of *MEX3A* depletion to suppress GB cell growth *in vitro*, we expected that the inhibition of MEX3A might affect GB growth also *in vivo*. To this purpose, GB primary cells, genetically silenced for *MEX3A* and overexpressing a luciferase reporter, were injected into the brain of NOD/SCID gamma (NSG) mice. Based on *in vitro* results showing superior efficacy in silencing *MEX3A* and inhibiting GB6 cell growth, the shMEX3A#2 construct was selected for the *in vivo* experiment. Animals that received *MEX3A*-depleted cells showed a reduced tumor size (**Figures 5A, B**) and a longer OS compared to control mice (**Figure 5C**). Consistent with the *in vitro* data, the knockdown of *MEX3A* was also associated to a significantly decreased expression of the proliferation marker Ki67 and a reduction of the stemness marker SOX2 (**Figures 5D–G**). Overall, these findings validate the efficacy of MEX3A inhibition as a promising strategy for GB treatment together with its diagnostic and prognostic potential, thereby confirming its translational significance in clinical settings.

## Discussion

Gliomas encompass a range of malignancies with varying degrees of aggression and clinical outcomes (2–4). These tumors are classified based on their histological features and molecular characteristics that significantly impact their treatment and prognosis (4, 40). The challenge in managing gliomas lies in their heterogeneity and the complexity of their biological behavior, which can lead to variable responses to treatment and difficulties in predicting patient outcomes (41).

WHO 2021 classification introduced novel molecular markers with subtype-specific expression patterns in gliomas, facilitating refined classification (4, 40). However, it is noteworthy that not all laboratories possess the capability to analyze an extensive array of specific genetic markers, thereby constraining their prognostic utility (15, 42). Investigating the molecular mechanisms involved in gliomas progression and recurrence represents a great opportunity to identify novel, straightforward, diagnostic and prognostic markers, and to unveil the intricate facets of gliomas tumor biology (43, 44).

MEX3A plays a crucial role in self-renewal and differentiation processes affecting stemness and carcinogenesis (17, 18, 21, 38). Acting through its RNA-binding and E3 ubiquitin ligase domains, MEX3A appears to influence several pathways relevant to tumor progression and therapy resistance. Notably, MEX3A has been shown to regulate the stability of key transcripts involved in cell cycle control and apoptosis, such as E2F and G2/M checkpoint targets (17, 21).

In glioma, MEX3A upregulates CCL2, a chemokine known to support proliferation, angiogenesis, and immune evasion, leading to alteration of the tumor microenvironment (TME) and promoting tumorigenesis (23). Moreover, MEX3A downregulates mRNA levels of *MSH2*, a key DNA mismatch repair (MMR) component, thereby impairing DNA repair fidelity and enhancing resistance to TMZ in GB patients (27). Indeed, as demonstrated by Gan and colleagues, MEX3A levels, especially in the context of MGMT promoter hypermethylation, may serve as a predictive marker for TMZ response. In this study, they showed that blocking MEX3A makes GB cells more sensitive to treatment by restoring MMR function (27).These findings, highlight the need to fully elucidate the role of MEX3A in glioma pathogenesis. In this regard, we discovered MEX3A as a new diagnostic and independent prognostic biomarker for GB making promising advancement in the field of glioma research. Indeed, MEX3A may provide several potential benefits that could enhance diagnostic and prognostic accuracy compared to currently used biomarkers (45–48). Firstly, MEX3A is highly expressed across all subtypes of gliomas, providing a reliable indicator of disease presence and progression. Secondly, MEX3A offers independent prognostic value associating with both OS and PFS in GB, providing clinicians with valuable insights into patient outcomes. Thirdly, our multiparametric analysis confirms that the prognostic impact of MEX3A is not influenced by commonly assessed markers such as EGFR, p53, or Ki67, highlighting its unique and standalone value. Finally, MEX3A is relatively easy to analyze, making it accessible to a wider range of laboratories and healthcare facilities. MEX3A analysis could be integrated into routine diagnostic workflows with minimal additional resources. Overall, our findings align with the growing body of research identifying biomarkers with diagnostic and prognostic significance in gliomas (49–57), further reinforcing the relevance of MEX3A in this context.

Noteworthy, we found that the genetic depletion of *MEX3A* inhibits the growth of primary GB cell both *in vitro* and *vivo*. These findings are consistent with the data reported in our previous study, in which we described for the first time, the role of MEX3A in GB growth (26). Interestingly, beyond its role in RNA regulation, our recent data highlight a previously unrecognized function of MEX3A as an E3 ubiquitin ligase involved in GB (26). We demonstrated that MEX3A promotes ubiquitylation and degradation of the retinoic acid inducible gene I (RIG-I) (26), an important pattern recognition receptor that acts as an RNA cytoplasmic sensor to activate the innate immune response and cell death via apoptosis (58–62). Interestingly, we showed that the genetic depletion of *MEX3A* leads to an increase in the RIG-I protein level and strongly inhibits the proliferation of GB cells (26). These evidence suggested the potential therapeutic implication of MEX3A in GB, either by its directly targeting or exploiting the functions of RIG-I. Indeed, this receptor detects viral single or double-strand RNA and once activated, triggers signaling pathways converging on the production of type I interferons, proinflammatory cytokines, and programmed cell death (59–63). Approaches aimed at activating RIG-I within cancers are being explored as novel therapeutic strategies to generate an inflammatory tumor microenvironment and to facilitate cytotoxic T-cell cross-priming and infiltration (63, 64). Recent evidence has shown that the activation of RIG-I promotes apoptosis in gliomas and increases the production of IFN-β and CXCL10, thereby inhibiting the tumor growth in both *in vitro* and *in vivo* models (65). Currently, ongoing Phase 1 and 2 clinical trials are investigating the efficacy of RIG-I agonists in several tumor types, including gliomas, indicating the potential versatility of these therapeutic agents in inducing cell death and modulating cytokines (63, 64). In the light of these evidence, new therapeutic approaches may arise from the targeting of both MEX3A and RIG-I using inhibitors able to block MEX3A functions and impair its interaction with RIG-I, as well as by the direct stimulation of RIG-I itself.

Further studies are needed for the better understanding the role of MEX3A and RIG-1 circuitry in gliomas pathogenesis, including the heterogeneity of the stem cells in gliomas (GSCs) (66). GSCs represent a highly diverse and plastic population of cells that exhibit dynamic stemness states, regulated by both intrinsic (genetic/epigenetic) and extrinsic (microenvironmental) factors (66). Multiple GSC subpopulations have been defined based on markers, such as CD133, CD44, SOX2; however, no single marker reliably identifies all GSCs, highlighting the inherent complexity of effectively targeting this cellular compartment (66, 67). Moreover, GSCs align with specific GB transcriptional subtypes, such as proneural, mesenchymal, and classical, each displaying unique molecular signatures, spatial distributions, and immunological profiles (66–68). Importantly, GSCs are not static but can interconvert with non-GSCs in response to environmental pressures, including therapy, contributing to tumor recurrence and treatment resistance (66–69). This cellular plasticity is further controlled by epigenetic mechanisms and the ubiquitin-proteasome system, suggesting that post-transcriptional modulators like MEX3A may play a pivotal role in this dynamic regulation. The TME, especially the immune component, also significantly affects GSCs behavior (68–70). GSCs actively shape the TME by promoting immunosuppression via the secretion of IL-10, TGF-β, and recruitment of regulatory T cells, while resisting immune clearance through upregulation of PD-L1 and modulation of antigen presentation (68–70). Notably, the interaction of MEX3A with RIG-I, a key activator of the innate immune response, may interfere with these processes. Thus, modulating MEX3A levels could not only impact tumor proliferation but also reshape the immune landscape of gliomas. Although a more comprehensive phenotypic characterization of GB-derived neurospheres would enhance the interpretation of our findings, this study highlights the important role of MEX3A in the maintaining stemness features of GB cells. In conclusion, targeting MEX3A, either alone or in combination with RIG-I activation, represents a compelling therapeutic strategy that could disrupt GSC-driven resistance, restore anti-tumor immunity, and ultimately shift the treatment paradigm in GB.

### Further studies and limitations

This study has some limitations that should be acknowledged. First, the retrospective design inherently limits control over confounding variables and may introduce selection bias. Second, being a single-center cohort, the findings might not be fully generalizable to broader or more diverse patient populations. Finally, the absence of an independent external validation set restricts the strength of the conclusions, and future multicenter prospective studies are necessary to confirm these results. Although future works are needed for the translation of this multi-targeting strategy in clinical practice, the present study could represent an innovative opportunity for the treatment of GB.

## Data Availability

The raw data supporting the conclusions of this article will be made available by the authors, without undue reservation.

## Supplementary material

Supplementary Figure 1 The flow-chart of selection of the final cohort of patients according to STROBE criteria.

Supplementary Figure 2
*MEX3A* mRNA expression according to different clinical and molecular parameters in glioma patients (n=81). **(A)** Age. 64 years is the median age of the patients at diagnosis. **(B)** Sex. **(C)** Grade. **(D)** Vital Status. **(E)** *IDH1* R132H mutation. **(F)** EGFR protein expression. **(G)** P53 protein expression. **(H)** %Ki67 protein expression. 20% is the median value of expression in the cohort of patients. Mean ± SD. ***P *<* 0.05.

Supplementary Figure 3 Diagnostic and prognostic value of MEX3A in *IDH*-wildtype GB. **(A)** MEX3A protein expression in 51 cases of GB compared to 14 peritumoral tissues (PTs). Mean ± SD; ***** P *<* 0.001. **(B)** ROC curve for MEX3A protein expression in GB and PTs shown in A. AUC= 0.8291. **(C, D)** Kaplan-Meyer curves for OS **(C)** and PFS **(D)** between MEX3A high and low protein expression groups.

Supplementary Figure 4 Characteristics of primary GB-derived neurospheres. Immuunoblot analysis of the indicated proteins in peritumoral tissues (PT), tumor tissues (TT) and the correspondent primary GB-derived neurospheres (PGN). Actin was used as loading control.

Supplementary Figure 5 Effect of *MEX3A* silencing on invasion and adhesion markers in GB-derived neurospheres. Representative immunoblot blot analysis of Vimentin and E-Cadherin in GB2 **(A)**, GB6 **(B)** and GB12 **(C)** GB-derived neurospheres following infection with lentiviral particles encoding either control shRNA (shCTR) or MEX3A shRNAs (shMEX3A#1 and shMEX3A#2). Actin was used as loading control.

## References

[1] Sonkin D Thomas A Teicher BA. Cancer treatments: Past, present, and future. Cancer Genet. (2024) 286–287:18–24. doi: 10.1016/j.cancergen.2024.06.002, PMID: 38909530 PMC11338712

[2] Ostrom QT Price M Neff C Cioffi G Waite KA Kruchko C. CBTRUS statistical report: primary brain and other central nervous system tumors diagnosed in the United States in 2016—2020. Neuro-Oncol. (2023) 25:iv1–99. doi: 10.1093/neuonc/noad149, PMID: 37793125 PMC10550277

[3] Price M Ballard C Benedetti J Neff C Cioffi G Waite KA. CBTRUS statistical report: primary brain and other central nervous system tumors diagnosed in the United States in 2017–2021. Neuro-Oncol. (2024) 26:vi1–85. doi: 10.1093/neuonc/noae145, PMID: 39371035 PMC11456825

[4] Louis DN Perry A Wesseling P Brat DJ Cree IA Figarella-Branger D. The 2021 WHO classification of tumors of the central nervous system: a summary. Neuro-Oncol. (2021) 23:1231–51. doi: 10.1093/neuonc/noab106, PMID: 34185076 PMC8328013

[5] Bray F Laversanne M Sung H Ferlay J Siegel RL Soerjomataram I. Global cancer statistics 2022: GLOBOCAN estimates of incidence and mortality worldwide for 36 cancers in 185 countries. CA Cancer J Clin. (2024) 74:229–63. doi: 10.3322/caac.21834, PMID: 38572751

[6] Manake R Phillips V Gangi A Ravikumar J. Trends in the incidence of brain cancer: an observational study. Cureus. (2024). 16:e72805. doi: 10.7759/cureus.72805, PMID: 39618616 PMC11608382

[7] Stupp R Mason WP Van Den Bent MJ Weller M Fisher B Taphoorn MJB. Radiotherapy plus concomitant and adjuvant temozolomide for glioblastoma. N Engl J Med. (2005) 352:987–96. doi: 10.1056/NEJMoa043330, PMID: 15758009

[8] Ringel F Pape H Sabel M Krex D Bock HC Misch M. Clinical benefit from resection of recurrent glioblastomas: results of a multicenter study including 503 patients with recurrent glioblastomas undergoing surgical resection. Neuro-Oncol. (2016) 18:96–104. doi: 10.1093/neuonc/nov145, PMID: 26243790 PMC4677413

[9] Weller M Van Den Bent M Preusser M Le Rhun E Tonn JC Minniti G. EANO guidelines on the diagnosis and treatment of diffuse gliomas of adulthood. Nat Rev Clin Oncol. (2021) 18:170–86. doi: 10.1038/s41571-020-00447-z, PMID: 33293629 PMC7904519

[10] Parsons DW Jones S Zhang X Lin JCH Leary RJ Angenendt P. An integrated genomic analysis of human glioblastoma multiforme. Science. (2008) 321:1807–12. doi: 10.1126/science.1164382, PMID: 18772396 PMC2820389

[11] Silantyev A Falzone L Libra M Gurina O Kardashova K Nikolouzakis T. Current and future trends on diagnosis and prognosis of glioblastoma: from molecular biology to proteomics. Cells. (2019) 8:863. doi: 10.3390/cells8080863, PMID: 31405017 PMC6721640

[12] Komori T. The 2021 WHO classification of tumors, 5th edition, central nervous system tumors: the 10 basic principles. Brain Tumor Pathol. (2022) 39:47–50. doi: 10.1007/s10014-022-00428-3, PMID: 35316415

[13] Szopa W Burley TA Kramer-Marek G Kaspera W. Diagnostic and therapeutic biomarkers in glioblastoma: current status and future perspectives. BioMed Res Int. (2017) 2017:1–13. doi: 10.1155/2017/8013575, PMID: 28316990 PMC5337853

[14] Bou Zerdan M Atoui A Hijazi A Basbous L Abou Zeidane R Alame SM. Latest updates on cellular and molecular biomarkers of gliomas. Front Oncol. (2022) 12:1030366. doi: 10.3389/fonc.2022.1030366, PMID: 36425564 PMC9678906

[15] Jain KK. A critical overview of targeted therapies for glioblastoma. Front Oncol. (2018) 8:419. doi: 10.3389/fonc.2018.00419, PMID: 30374421 PMC6196260

[16] Ernster AE Klepin HD Lesser GJ. Strategies to assess and manage frailty among patients diagnosed with primary Malignant brain tumors. Curr Treat Options Oncol. (2024) 25:27–41. doi: 10.1007/s11864-023-01167-z, PMID: 38194149 PMC11298213

[17] Jasinski-Bergner S Steven A Seliger B. The role of the RNA-binding protein family MEX-3 in tumorigenesis. Int J Mol Sci. (2020) 21:5209. doi: 10.3390/ijms21155209, PMID: 32717840 PMC7432607

[18] Pereira B Le Borgne M Chartier NT Billaud M Almeida R. MEX-3 proteins: recent insights on novel post-transcriptional regulators. Trends Biochem Sci. (2013) 38:477–9. doi: 10.1016/j.tibs.2013.08.004, PMID: 23999169

[19] Liang J Li H Han J Jiang J Wang J Li Y. Mex3a interacts with LAMA2 to promote lung adenocarcinoma metastasis via PI3K/AKT pathway. Cell Death Dis. (2020) 11:614. doi: 10.1038/s41419-020-02858-3, PMID: 32792503 PMC7427100

[20] Yang D Jiao Y Li Y Fang X. Clinical characteristics and prognostic value of MEX3A mRNA in liver cancer. PeerJ. (2020) 8:e8252. doi: 10.7717/peerj.8252, PMID: 31998552 PMC6979405

[21] Lederer M Müller S Glaß M Bley N Ihling C Sinz A. Oncogenic potential of the dual-function protein MEX3A. Biology. (2021) 10:415. doi: 10.3390/biology10050415, PMID: 34067172 PMC8151450

[22] Panzeri V Manni I Capone A Naro C Sacconi A Di Agostino S. The RNA-binding protein MEX3A is a prognostic factor and regulator of resistance to gemcitabine in pancreatic ductal adenocarcinoma. Mol Oncol. (2021) 15:579–95. doi: 10.1002/1878-0261.12847, PMID: 33159833 PMC7858117

[23] Yang C Zhan H Zhao Y Wu Y Li L Wang H. MEX3A contributes to development and progression of glioma through regulating cell proliferation and cell migration and targeting CCL2. Cell Death Dis. (2021) 12:14. doi: 10.1038/s41419-020-03307-x, PMID: 33414423 PMC7791131

[24] Li H Liang J Wang J Han J Li S Huang K. Mex3a promotes oncogenesis through the RAP1/MAPK signaling pathway in colorectal cancer and is inhibited by hsa-miR-6887-3p. Cancer Commun. (2021) 41:472–91. doi: 10.1002/cac2.12149, PMID: 33638620 PMC8211350

[25] Li F Zhao C Diao Y Wang Z Peng J Yang N. MEX3A promotes the Malignant progression of ovarian cancer by regulating intron retention in TIMELESS. Cell Death Dis. (2022) 13:553. doi: 10.1038/s41419-022-05000-7, PMID: 35715407 PMC9205863

[26] Bufalieri F Caimano M Lospinoso Severini L Basili I Paglia F Sampirisi L. The RNA-binding ubiquitin ligase MEX3A affects glioblastoma tumorigenesis by inducing ubiquitylation and degradation of RIG-I. Cancers. (2020) 12:321. doi: 10.3390/cancers12020321, PMID: 32019099 PMC7072305

[27] Gan T Wang Y Xie M Wang Q Zhao S Wang P. MEX3A impairs DNA mismatch repair signaling and mediates acquired temozolomide resistance in glioblastoma. Cancer Res. (2022) 82:4234–46. doi: 10.1158/0008-5472.CAN-22-2036, PMID: 36112059

[28] The Gene Ontology Consortium. The Gene Ontology Resource: 20 years and still GOing strong. Nucleic Acids Res. (2019) 47:D330–8. doi: 10.1093/nar/gky1055, PMID: 30395331 PMC6323945

[29] Armocida D Pesce A Palmieri M D’Andrea G Salvati M Santoro A. Periventricular zone involvement as a predictor of survival in glioblastoma patients: A single centre cohort-comparison investigation concerning a distinct clinical entity. Interdiscip Neurosurg. (2021) :25:101185. doi: 10.1016/j.inat.2021.101185

[30] Pesce A Armocida D Paglia F Palmieri M Frati A D’Andrea G. IDH wild-type glioblastoma presenting with seizure: clinical specificity, and oncologic and surgical outcomes. J Neurol Surg Part Cent Eur Neurosurg. (2022) 83:351–60. doi: 10.1055/s-0041-1735515, PMID: 34794192

[31] Lindboe CF Torp SH. Comparison of Ki-67 equivalent antibodies. J Clin Pathol. (2002) 55:467–71. doi: 10.1136/jcp.55.6.467, PMID: 12037032 PMC1769671

[32] Nagpal J Jamoona A Gulati ND Mohan A Braun A Murali R. Revisiting the role of p53 in primary and secondary glioblastomas. Anticancer Res. (2006) 26:4633–9., PMID: 17214319

[33] Leung SCY Nielsen TO Zabaglo L Arun I Badve SS Bane AL. Analytical validation of a standardized scoring protocol for Ki67: phase 3 of an international multicenter collaboration. NPJ Breast Cancer. (2016) 2:16014. doi: 10.1038/npjbcancer.2016.14, PMID: 28721378 PMC5515324

[34] Hazra A Gogtay N. Biostatistics series module 4: comparing groups – categorical variables. Indian J Dermatol. (2016) 61:385–92. doi: 10.4103/0019-5154.185700, PMID: 27512183 PMC4966396

[35] Smeltzer MP Ray MA. Statistical considerations for outcomes in clinical research: A review of common data types and methodology. Exp Biol Med. (2022) 247:734–42. doi: 10.1177/15353702221085710, PMID: 35492022 PMC9134761

[36] Lee J Kotliarova S Kotliarov Y Li A Su Q Donin NM. Tumor stem cells derived from glioblastomas cultured in bFGF and EGF more closely mirror the phenotype and genotype of primary tumors than do serum-cultured cell lines. Cancer Cell. (2006) 9:391–403. doi: 10.1016/j.ccr.2006.03.030, PMID: 16697959

[37] Da Hora CC Schweiger MW Wurdinger T Tannous BA. Patient-derived glioma models: from patients to dish to animals. Cells. (2019) 8:1177. doi: 10.3390/cells8101177, PMID: 31574953 PMC6829406

[38] Naef V De Sarlo M Testa G Corsinovi D Azzarelli R Borello U. The stemness gene mex3A is a key regulator of neuroblast proliferation during neurogenesis. Front Cell Dev Biol. (2020) :8:549533. doi: 10.3389/fcell.2020.549533, PMID: 33072742 PMC7536324

[39] Domingo-Muelas A Duart-Abadia P Morante-Redolat JM Jordán-Pla A Belenguer G Fabra-Beser J. Post-transcriptional control of a stemness signature by RNA-binding protein MEX3A regulates murine adult neurogenesis. Nat Commun. (2023) 14:373. doi: 10.1038/s41467-023-36054-6, PMID: 36690670 PMC9871011

[40] Armocida D Pesce A Santoro A Salvati M Frati A. Letter to the editor: “The neurosurgical perspective for the 2021 WHO classification of tumors of the central nervous system: A missed opportunity? World Neurosurg. (2021) 155:203–4. doi: 10.1016/j.wneu.2021.07.149, PMID: 34724739

[41] Fernández C Zafra-Martín J Couñago F. Current challenges in the treatment of gliomas: The molecular era. World J Clin Oncol. (2024) 15:982–6. doi: 10.5306/wjco.v15.i8.982, PMID: 39193161 PMC11346069

[42] Nakasu S Deguchi S Nakasu Y. IDH wild-type lower-grade gliomas with glioblastoma molecular features: a systematic review and meta-analysis. Brain Tumor Pathol. (2023) 40:143–57. doi: 10.1007/s10014-023-00463-8, PMID: 37212969

[43] Le Rhun E Preusser M Roth P Reardon DA van den Bent M Wen P. Molecular targeted therapy of glioblastoma. Cancer Treat Rev. (2019) 80:101896. doi: 10.1016/j.ctrv.2019.101896, PMID: 31541850

[44] Balana C Castañer S Carrato C Moran T Lopez-Paradís A Domenech M. Preoperative diagnosis and molecular characterization of gliomas with liquid biopsy and radiogenomics. Front Neurol. (2022) 13:865171. doi: 10.3389/fneur.2022.865171, PMID: 35693015 PMC9177999

[45] Kan LK Drummond K Hunn M Williams D O’Brien TJ Monif M. Potential biomarkers and challenges in glioma diagnosis, therapy and prognosis. BMJ Neurol Open. (2020) 2:e000069. doi: 10.1136/bmjno-2020-000069, PMID: 33681797 PMC7871709

[46] Armocida D Pesce A Frati A Santoro A Salvati M. EGFR amplification is a real independent prognostic impact factor between young adults and adults over 45yo with wild-type glioblastoma? J Neurooncol. (2020) 146:275–84. doi: 10.1007/s11060-019-03364-z, PMID: 31889239

[47] Erira A Velandia F Penagos J Zubieta C Arboleda G. Differential regulation of the EGFR/PI3K/AKT/PTEN pathway between low- and high-grade gliomas. Brain Sci. (2021) 11:1655. doi: 10.3390/brainsci11121655, PMID: 40536529 PMC12178274

[48] Sareen H Ma Y Becker TM Roberts TL De Souza P Powter B. Molecular biomarkers in glioblastoma: A systematic review and meta-analysis. Int J Mol Sci. (2022) 23:8835. doi: 10.3390/ijms23168835, PMID: 36012105 PMC9408540

[49] Lié O Virolle T Gabut M Pasquier C Zemmoura I Augé-Gouillou C. SETMAR shorter isoform: A new prognostic factor in glioblastoma. Front Oncol. (2022) 11:638397. doi: 10.3389/fonc.2021.638397, PMID: 35047379 PMC8761672

[50] Ma X Geng R Zhao Y Xu W Li Y Jiang Y. CHRNA9 as a new prognostic marker and potential therapeutic target in glioma. J Cancer. (2024) 15:2095–109. doi: 10.7150/jca.92080, PMID: 38495483 PMC10937273

[51] Fan Y Yan D Ma L Liu X Luo G Hu Y. ALKBH5 is a prognostic factor and promotes the angiogenesis of glioblastoma. Sci Rep. (2024) 14:1303. doi: 10.1038/s41598-024-51994-9, PMID: 38221546 PMC10788339

[52] He W Zhang Z Tan Z Liu X Wang Z Xiong B. PSMB2 plays an oncogenic role in glioma and correlates to the immune microenvironment. Sci Rep. (2024) 14:5861. doi: 10.1038/s41598-024-56493-5, PMID: 38467767 PMC10928079

[53] Wang Z Wang D Wang X Xu Y Yuan Y Chen Y. Integrative analysis of SEPN1 in glioma: Prognostic roles, functional implications, and potential therapeutic interventions. PloS One. (2025) 20:e0318501. doi: 10.1371/journal.pone.0318501, PMID: 39919065 PMC11805447

[54] Liu H Weng J. A comprehensive bioinformatic analysis of cyclin-dependent kinase 2 (CDK2) in glioma. Gene. (2022) 822:146325. doi: 10.1016/j.gene.2022.146325, PMID: 35183683

[55] Liu H Tang T. A bioinformatic study of IGFBPs in glioma regarding their diagnostic, prognostic, and therapeutic prediction value. Am J Transl Res. (2023) 15:2140–55., PMID: 37056850 PMC10086936

[56] Liu H Tang T. MAPK signaling pathway-based glioma subtypes, machine-learning risk model, and key hub proteins identification. Sci Rep. (2023) 13:19055. doi: 10.1038/s41598-023-45774-0, PMID: 37925483 PMC10625624

[57] Liu H Hamaia SW Dobson L Weng J Hernández FL Beaudoin CA. The voltage-gated sodium channel β3 subunit modulates C6 glioma cell motility independently of channel activity. Biochim Biophys Acta BBA - Mol Basis Dis. (2025) 1871:167844. doi: 10.1016/j.bbadis.2025.167844, PMID: 40245999

[58] Yoneyama M Kikuchi M Natsukawa T Shinobu N Imaizumi T Miyagishi M. The RNA helicase RIG-I has an essential function in double-stranded RNA-induced innate antiviral responses. Nat Immunol. (2004) 5:730–7. doi: 10.1038/ni1087, PMID: 15208624

[59] Wu Y Wu X Wu L Wang X Liu Z. The anticancer functions of RIG-I–like receptors, RIG-I and MDA5, and their applications in cancer therapy. Transl Res. (2017) 190:51–60. doi: 10.1016/j.trsl.2017.08.004, PMID: 28917654

[60] Xu Xx Wan H Nie L Shao T Xiang Lx Shao Jz. RIG-I: a multifunctional protein beyond a pattern recognition receptor. Protein Cell. (2018) 9:246–53. doi: 10.1007/s13238-017-0431-5, PMID: 28593618 PMC5829270

[61] Rehwinkel J Gack MU. RIG-I-like receptors: their regulation and roles in RNA sensing. Nat Rev Immunol. (2020) 20:537–51. doi: 10.1038/s41577-020-0288-3, PMID: 32203325 PMC7094958

[62] Thoresen D Wang W Galls D Guo R Xu L Pyle AM. The molecular mechanism of RIG-I activation and signaling. Immunol Rev. (2021) 304:154–68. doi: 10.1111/imr.13022, PMID: 34514601 PMC9293153

[63] Elion DL Cook RS. Harnessing RIG-I and intrinsic immunity in the tumor microenvironment for therapeutic cancer treatment. Oncotarget. (2018) 9:29007–17. doi: 10.18632/oncotarget.25626, PMID: 29989043 PMC6034747

[64] Bufalieri F Basili I Di Marcotullio L Infante P. Harnessing the activation of RIG-I like receptors to inhibit glioblastoma tumorigenesis. Front Mol Neurosci. (2021) 14:710171. doi: 10.3389/fnmol.2021.710171, PMID: 34305530 PMC8295747

[65] Chen J Liu Z Fang H Su Q Fan Y Song L. Therapeutic efficacy of a novel self-assembled immunostimulatory siRNA combining apoptosis promotion with RIG-I activation in gliomas. J Transl Med. (2024) 22:395. doi: 10.1186/s12967-024-05151-5, PMID: 38685028 PMC11057130

[66] Silver A Feier D Ghosh T Rahman M Huang J Sarkisian MR. Heterogeneity of glioblastoma stem cells in the context of the immune microenvironment and geospatial organization. Front Oncol. (2022) 12:1022716. doi: 10.3389/fonc.2022.1022716, PMID: 36338705 PMC9628999

[67] Thakkar JP Dolecek TA Horbinski C Ostrom QT Lightner DD Barnholtz-Sloan JS. Epidemiologic and molecular prognostic review of glioblastoma. Cancer Epidemiol Biomarkers Prev. (2014) 23:1985–96. doi: 10.1158/1055-9965.EPI-14-0275, PMID: 25053711 PMC4185005

[68] Piper K DePledge L Karsy M Cobbs C. Glioma stem cells as immunotherapeutic targets: advancements and challenges. Front Oncol. (2021) 11:615704. doi: 10.3389/fonc.2021.615704, PMID: 33718170 PMC7945033

[69] Gimple RC Bhargava S Dixit D Rich JN. Glioblastoma stem cells: lessons from the tumor hierarchy in a lethal cancer. Genes Dev. (2019) 33:591–609. doi: 10.1101/gad.324301.119, PMID: 31160393 PMC6546059

[70] Johnson AL Laterra J Lopez-Bertoni H. Exploring glioblastoma stem cell heterogeneity: Immune microenvironment modulation and therapeutic opportunities. Front Oncol. (2022) 12:995498. doi: 10.3389/fonc.2022.995498, PMID: 36212415 PMC9532940

