# Supplementary figures and images for: MEX3A is a diagnostic, independent prognostic biomarker and a promising therapeutic target in glioblastoma
Source: Front Oncol. 2025 Sep 1;15:1585592. doi: 10.3389/fonc.2025.1585592 (PMC12433882; doi:10.3389/fonc.2025.1585592)

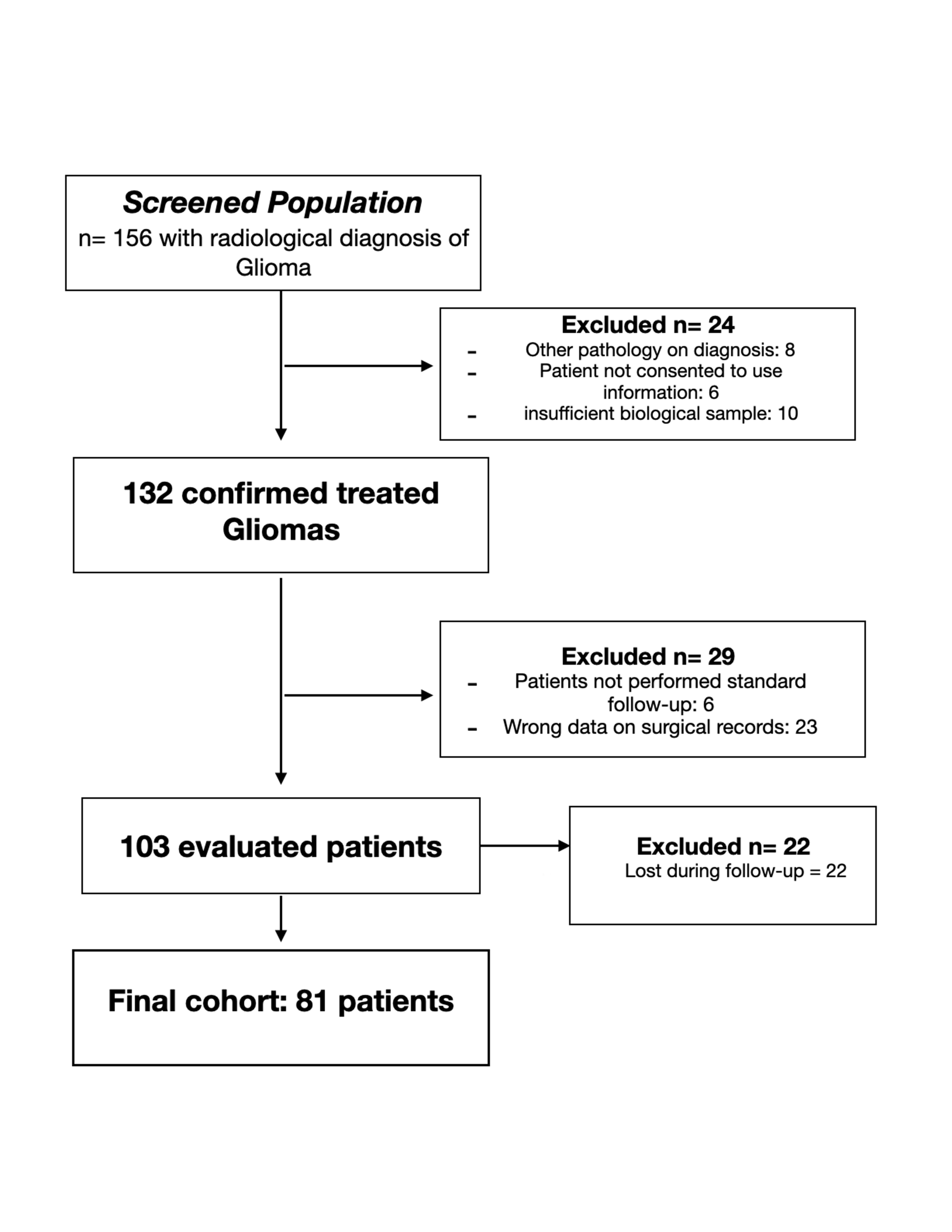

Supplement: Supplementary Figure 1 — The flow-chart of selection of the final cohort of patients according to STROBE criteria. [file Image1.jpeg]

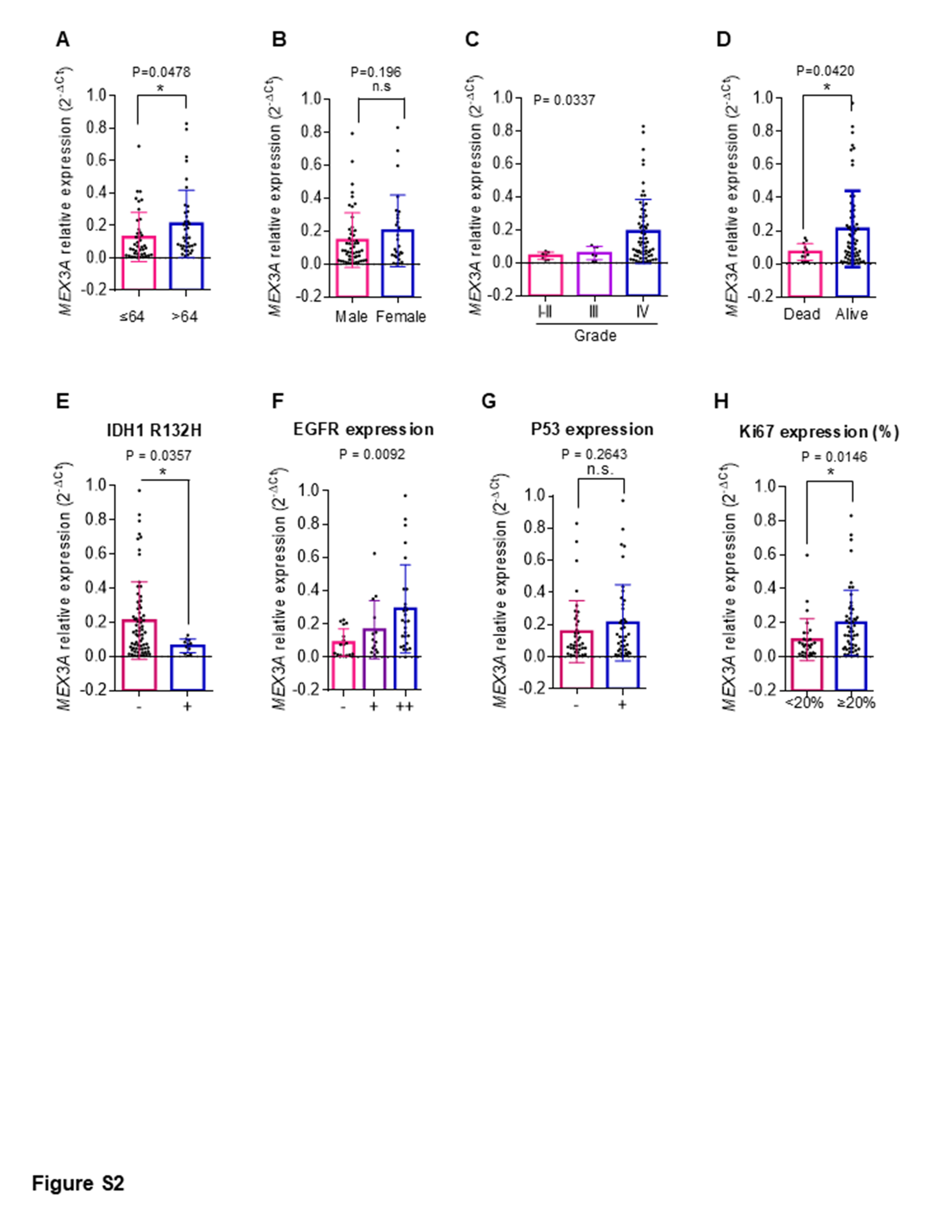

Supplement: Supplementary Figure 2 — MEX3A mRNA expression according to different clinical and molecular parameters in glioma patients (n=81). (A) Age. 64 years is the median age of the patients at diagnosis. (B) Sex. (C) Grade. (D) Vital Status. (E) IDH1 R132H mutation. (F) EGFR protein expression. (G) P53 protein expression. (H) %Ki67 protein expression. 20% is the median value of expression in the cohort of patients. Mean ± SD. *P < 0.05. [file Image2.jpg]

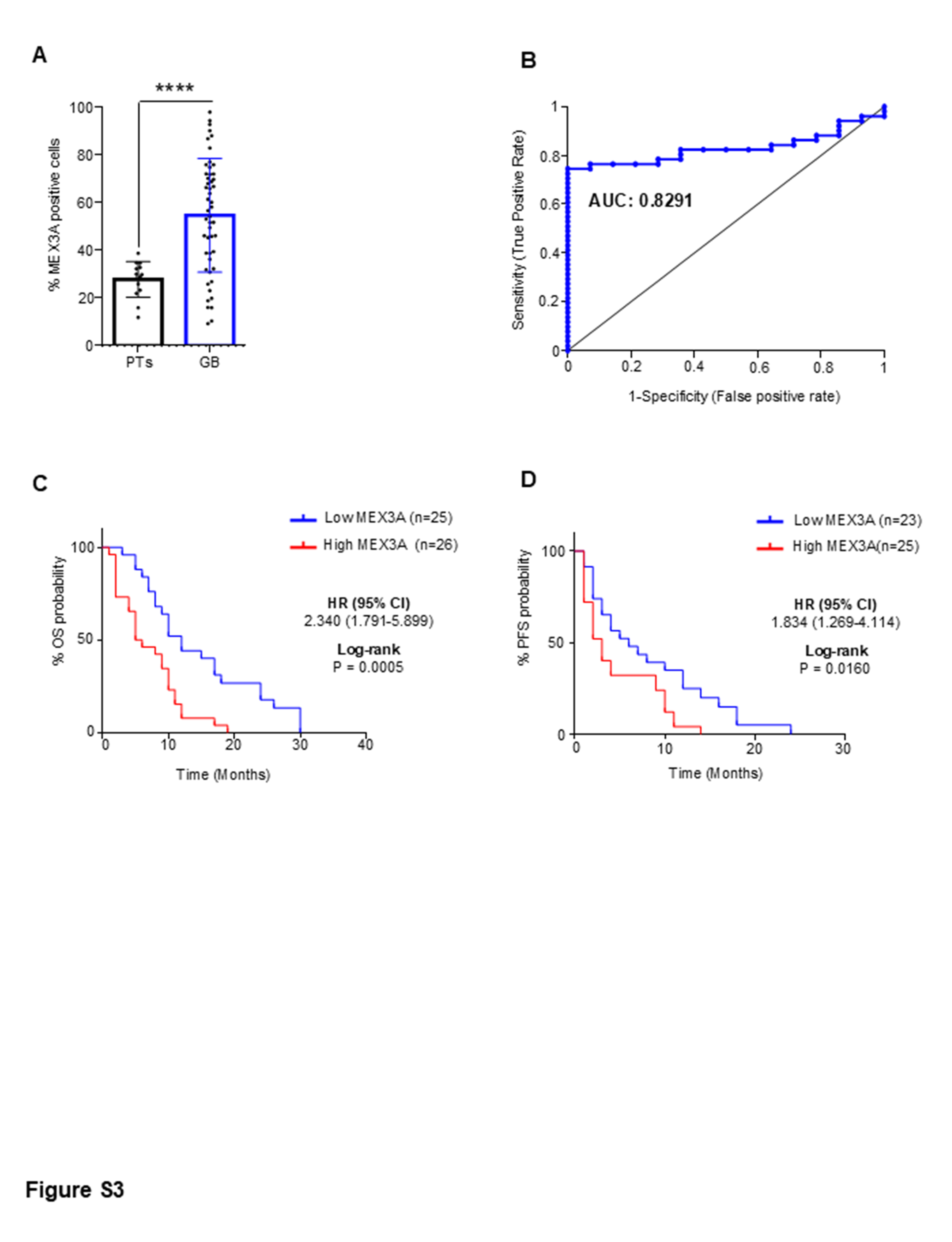

Supplement: Supplementary Figure 3 — Diagnostic and prognostic value of MEX3A in IDH-wildtype GB. (A) MEX3A protein expression in 51 cases of GB compared to 14 peritumoral tissues (PTs). Mean ± SD; *** P < 0.001. (B) ROC curve for MEX3A protein expression in GB and PTs shown in A. AUC= 0.8291. (C, D) Kaplan-Meyer curves for OS (C) and PFS (D) between MEX3A high and low protein expression groups. [file Image3.jpg]

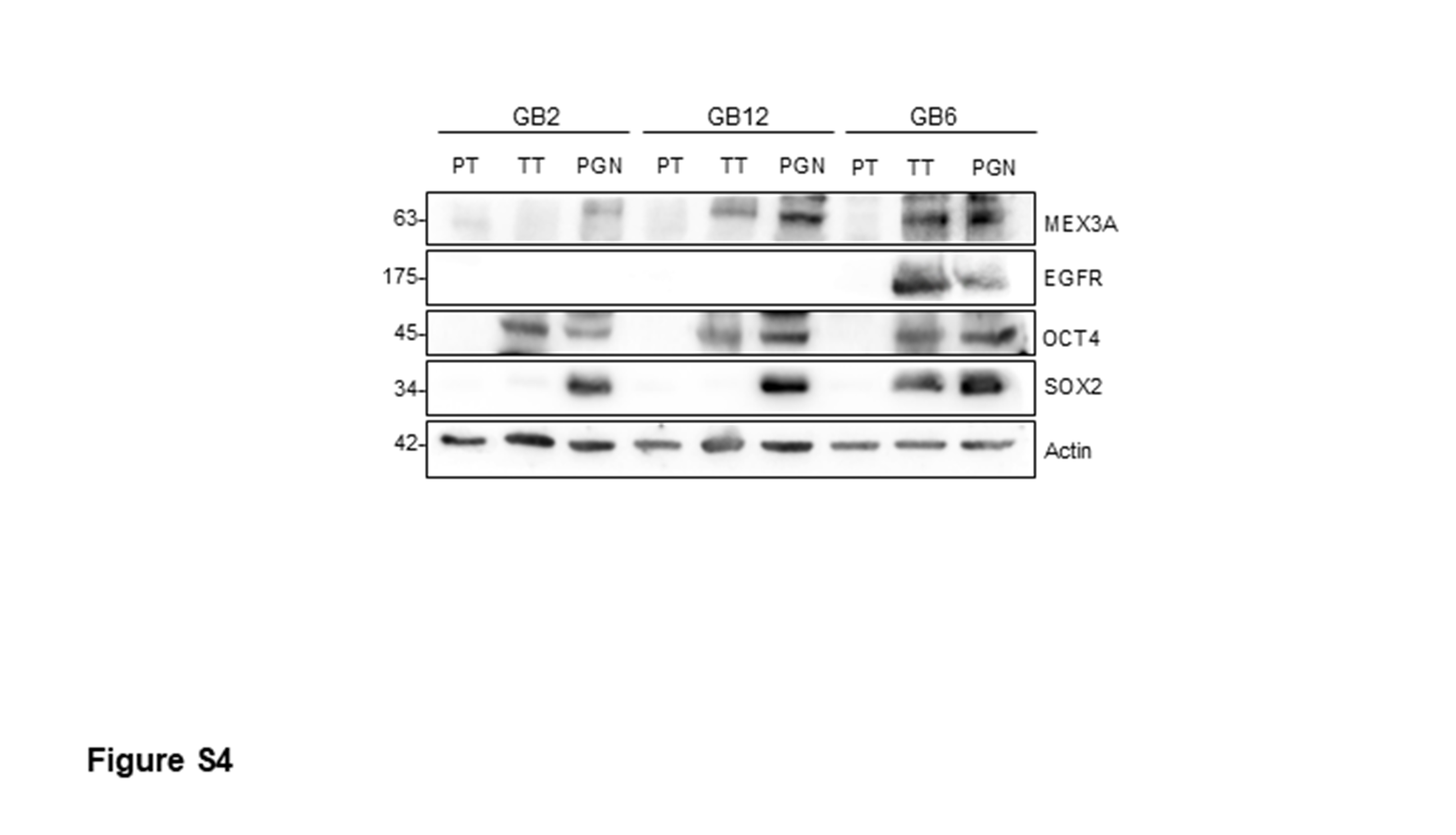

Supplement: Supplementary Figure 4 — Characteristics of primary GB-derived neurospheres. Immuunoblot analysis of the indicated proteins in peritumoral tissues (PT), tumor tissues (TT) and the correspondent primary GB-derived neurospheres (PGN). Actin was used as loading control. [file Image4.jpeg]

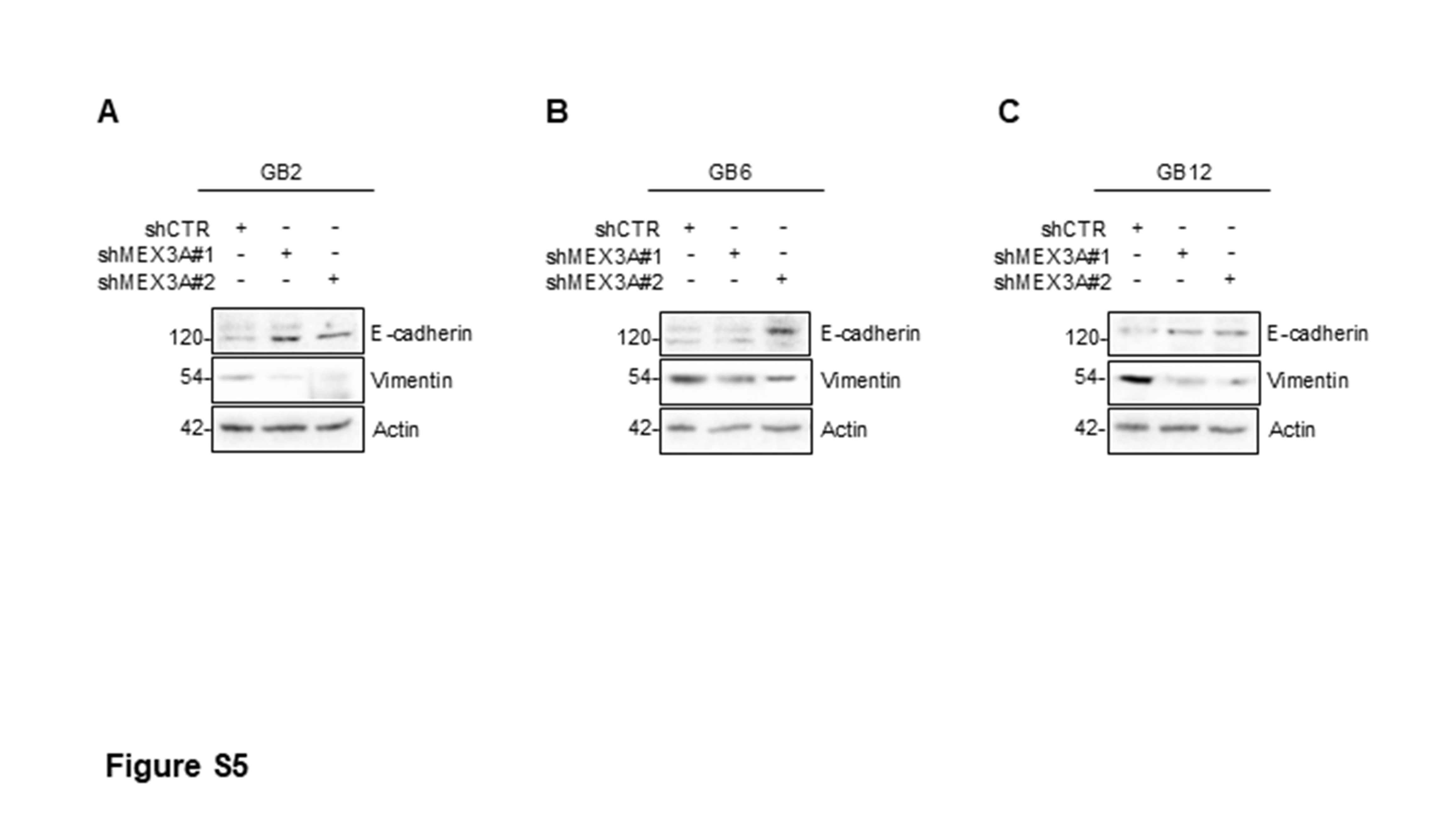

Supplement: Supplementary Figure 5 — Effect of MEX3A silencing on invasion and adhesion markers in GB-derived neurospheres. Representative immunoblot blot analysis of Vimentin and E-Cadherin in GB2 (A), GB6 (B) and GB12 (C) GB-derived neurospheres following infection with lentiviral particles encoding either control shRNA (shCTR) or MEX3A shRNAs (shMEX3A#1 and shMEX3A#2). Actin was used as loading control. [file Image5.jpeg]
