# Supplementary material for: MEX3A is a diagnostic, independent prognostic biomarker and a promising therapeutic target in glioblastoma
Source: Front Oncol. 2025 Sep 1;15:1585592. doi: 10.3389/fonc.2025.1585592 (PMC12433882; doi:10.3389/fonc.2025.1585592)
Supplement: Supplementary file 6 [file Table1.docx]

| Clinical characteristics | **Number of patients (n=81)** | % |
| --- | --- | --- |
| **Sex** | | |
| M | 53 | 65.4 |
| F | 28 | 34.6 |
| **Age** | | |
| Mean | 61.5 |  |
| Min. | 26 |  |
| Max | 84 |  |
| **Diagnosis** | | |
| Glioblastoma | 63 | 77.8 |
| Diffuse astrocytoma | 7 | 8.6 |
| Anaplastic astrocytoma | 4 | 4.9 |
| Oligodendroglioma | 4 | 4.9 |
| Gliosarcoma | 2 | 2.5 |
| PLNTY | 1 | 1.2 |
| **Grading** | | |
| 4 | 67 | 82.7 |
| 3 | 7 | 8.6 |
| 2 | 6 | 7.4 |
| 1 | 1 | 1.2 |
| **PFS** |  |  |
| Mean | 6.8 |  |
| Min. | 1 |  |
| Max | 53 |  |
| **No progression** | 9 | 11.11 |
| **OS** |  |  |
| Mean | 14.7 |  |
| Min. | 1 |  |
| Max | 60 |  |
| **Alive** | 12 | 14.8 |
| Molecular characteristics |  |  |
| IDH1 R132H | 11 | 13.6 |
| **EGFR expression** | 41 | 71.9 |
| P53 expression | 42 | 51.8 |
| **%Ki67 expression** |  |  |
| Mean | 22.4 |  |
| Min. | 1 |  |
| Max. | 50 |  |

Table S1. Clinic and molecular characteristics of the cohort of glioma patients
