# Supplementary material for: MEX3A is a diagnostic, independent prognostic biomarker and a promising therapeutic target in glioblastoma
Source: Front Oncol. 2025 Sep 1;15:1585592. doi: 10.3389/fonc.2025.1585592 (PMC12433882; doi:10.3389/fonc.2025.1585592)
Supplement: Supplementary file 7 [file Table2.docx]

Table S2. Cell line authentication test by STR DNA profiling of different primary gliomas cell line with the characteristic of the donor patients

| **Sample Name** | human GB line 2 | human GB line 6 | human GB line 12 |
| --- | --- | --- | --- |
| **Sample Code** | CL00015692 | CL00015694 | CL00015695 |
| **ASN-002 core markers** |  |  |  |
| D7S820 | 8,11,12 | 8,11 | 9,13 |
| CSF1PO | 12,13 | 12,12 | 13,13 |
| TH01 | 8,8 | 8,8 | 9,9 |
| D13S317 | 14,14 | 13,13 | 12,12 |
| D16S539 | 11,11 | 10,10 | 12,12 |
| vWA | 14,17,18 | 14,18,19 | 18,18 |
| TPOX | 8,8 | 8,8 | 10,10 |
| AMEL | X,X | X,X | X,Y |
| D5S818 | 11,11 | 11,12 | 10,10 |
|  | * | * | * |
| **Clinical and molecular characteristic of the donor patient** |  |  |  |
| Gender | Male | Female | Male |
| Age | 57 | 82 | 81 |
| Hystological Type | Glioblastoma | Glioblastoma | Glioblastoma |
| Survival Time (months) | 18 | 4 | 5 |
| Survival Status | Died | Died | Died |
| IDH1 (R132) | No | No | No |
| EGFR  expression | No | Yes | Yes |
| P53  expression | Yes | Yes | No |
| Ki67  expression (%) | 15 | 30 | 30 |

* Cell line is not present in DSMZ / Cellosaurus databases.
